# *Streptomyces* Endophytes Promote Host Health and Enhance Growth across Plant Species

**DOI:** 10.1128/AEM.01053-20

**Published:** 2020-08-03

**Authors:** Sarah F. Worsley, Jake Newitt, Johannes Rassbach, Sibyl F. D. Batey, Neil A. Holmes, J. Colin Murrell, Barrie Wilkinson, Matthew I. Hutchings

**Affiliations:** a School of Biological Sciences, University of East Anglia, Norwich, United Kingdom; b Department of Molecular Microbiology, John Innes Centre, Norwich, United Kingdom; c School of Environmental Sciences, University of East Anglia, Norwich, United Kingdom; University of Bayreuth

**Keywords:** *Arabidopsis*, plant-microbe interactions, *Streptomyces*, wheat

## Abstract

*Streptomyces* bacteria are ubiquitous in soils and are well known for producing secondary metabolites, including antimicrobials. Increasingly, they are being isolated from plant roots, and several studies have shown they are specifically recruited to the rhizosphere and the endosphere of the model plant Arabidopsis thaliana. Here, we test the hypothesis that *Streptomyces* bacteria have a beneficial effect on *A. thaliana* growth and could potentially be used as plant probiotics. To do this, we selectively isolated streptomycetes from surface-washed *A. thaliana* roots and generated high-quality genome sequences for five strains, which we named L2, M2, M3, N1, and N2. Reinfection of *A. thaliana* plants with L2, M2, and M3 significantly increased plant biomass individually and in combination, whereas N1 and N2 had a negative effect on plant growth, likely due to their production of polyene natural products which can bind to phytosterols and reduce plant growth. N2 exhibits broad-spectrum antimicrobial activity and makes filipin-like polyenes, including 14-hydroxyisochainin which inhibits the take-all fungus, Gaeumannomyces graminis var. *tritici*. N2 antifungal activity as a whole was upregulated ∼2-fold in response to indole-3-acetic acid (IAA), suggesting a possible role during competition in the rhizosphere. Furthermore, coating wheat seeds with N2 spores protected wheat seedlings against take-all disease. We conclude that at least some soil-dwelling streptomycetes confer growth-promoting benefits on *A. thaliana*, while others might be exploited to protect crops against disease.

**IMPORTANCE** We must reduce reliance on agrochemicals, and there is increasing interest in using bacterial strains to promote plant growth and protect against disease. Our study follows up reports that Arabidopsis thaliana specifically recruits *Streptomyces* bacteria to its roots. We test the hypotheses that they offer benefits to their *A. thaliana* hosts and that strains isolated from these plants might be used as probiotics. We isolated *Streptomyces* strains from *A. thaliana* roots and genome sequenced five phylogenetically distinct strains. Genome mining and bioassays indicated that all five have plant growth-promoting properties, including production of indole-3-acetic acid (IAA), siderophores, and aminocyclopropane-1-carboxylate (ACC) deaminase. Three strains significantly increased *A. thaliana* growth *in vitro* and in combination in soil. Another produces potent filipin-like antifungals and protected germinating wheat seeds against the fungal pathogen Gaeumannomyces graminis var. *tritici* (wheat take-all fungus). We conclude that introducing *Streptomyces* strains into the root microbiome provides significant benefits to plants.

## Abstract (precis)

We must reduce reliance on agrochemicals, and there is increasing interest in using bacterial strains to promote plant growth and protect against disease. Our study follows up reports that Arabidopsis thaliana specifically recruits *Streptomyces* bacteria to its roots. We test the hypotheses that they offer benefits to their *A. thaliana* hosts and that strains isolated from these plants might be used as probiotics. We isolated *Streptomyces* strains from *A. thaliana* roots and genome sequenced five phylogenetically distinct strains. Genome mining and bioassays indicated that all five have plant growth-promoting properties, including production of indole-3-acetic acid (IAA), siderophores, and aminocyclopropane-1-carboxylate (ACC) deaminase. Three strains significantly increased *A. thaliana* growth *in vitro* and in combination in soil. Another produces potent filipin-like antifungals and protected germinating wheat seeds against the fungal pathogen Gaeumannomyces graminis var. *tritici* (wheat take-all fungus). We conclude that introducing *Streptomyces* strains into the root microbiome provides significant benefits to plants.

## INTRODUCTION

The bacterial genus *Streptomyces* comprises more than 600 known species, and they produce a diverse array of specialized metabolites that account for ∼55% of the antibiotics currently used in human medicine (1). They are filamentous spore-forming bacteria that are ubiquitous in soils, where they play an important role in breaking down complex organic material (2, 3). Intriguingly, they only produce around 10% of their encoded secondary metabolites *in vitro* (2–4). Thus, understanding the role and regulation of their specialized metabolites in natural habitats is essential if we are to unlock the other 90% and discover new molecules (3). Increasingly, *Streptomyces* species are being recognized as important defensive symbionts of a wide range of invertebrate species, including bees, beetles, digger wasps, and ants (5–9). In addition to this, streptomycetes have also been shown to interact extensively with plant roots, inhabiting both the soil surrounding the plant root, called the rhizosphere, and the niche within and between root cells, referred to as the endophytic compartment (10, 11). It has even been suggested that their filamentous hyphal growth and complex specialized metabolism have evolved to facilitate interactions with plant roots, presumably to allow entry into root tissue and enable them to compete for food in the form of root exudates or more complex polymers that make up the plant cell wall (2).

Several recent studies have reported that streptomycetes are present, and sometimes enriched, in the endophytic compartment of the model plant Arabidopsis thaliana relative to that in the bulk soil (12–15), where they are attracted by plant metabolites in the root exudates such as salicylate and jasmonate (16, 17). They have also been isolated from the endospheres of many other plant species, including wheat, a crop of huge social and economic value (18–21). Due to their capacity to produce a large number of antimicrobial compounds and their ability to abundantly colonize plant roots, streptomycetes are gaining increasing interest from a biocontrol point of view (10, 11, 19, 22). A recent study demonstrated that certain strains can act as defensive mutualists of strawberry plants, whereby they protect their host plant and pollinating bees against fungal infections (23). Many other isolates have been shown to protect important crops against infection, and two strains have been developed into commercial biocontrol agents called Actinovate and Mycostop (10, 19, 22, 24). The ubiquity of streptomycetes in soil and their diverse specialized metabolism, combined with their ability to colonize plant roots, make streptomycetes attractive for this purpose. Their spore-forming capabilities also make them tolerant of many environmental pressures, allowing them to be applied as dried seed coatings which remain viable under various agricultural conditions.

The aim of this study was to test the hypotheses that plant-associated streptomycetes provide benefits to their host *A. thaliana* plant and that strains isolated from *A. thaliana* might confer benefits to important crop plants, such as wheat. We hypothesized that these strains may play defensive or plant growth-promoting roles, since this genus is consistently recruited to the plant root microbiome. To this end, we generated high-quality genome sequences for five *Streptomyces* species which we isolated from the root microbiome of *A. thaliana* plants and which could recolonize *A. thaliana* roots when applied as seed coatings. All five strains, named L2, M2, M3, N1, and N2, harbor a large number of secondary metabolite biosynthetic gene clusters (BGCs), and all strains inhibited at least one bacterial or fungal pathogen. Strain N2 demonstrated broad-spectrum antifungal and antibacterial activity and was able to inhibit growth of the take-all fungus, Gaeumannomyces graminis var. *tritici*, an economically important pathogen of wheat, both *in vitro* and on germinating wheat seeds. The antifungal activity of N2 was increased 2-fold *in vitro* in response to indole-3-acetic acid (IAA), and purification of the antifungal molecules identified a number of filipin-like compounds. Curiously, N2 reduced *A. thaliana* growth *in vitro* (but not in soil) which may have been caused by filipins targeting sterols in the plant cell membranes at high concentrations. Strains L2, M2, and M3 all promoted *A. thaliana* growth *in vitro* and when applied in combination to seeds planted in soil, and they all have well-characterized plant growth-promoting (PGP) traits, including the production of plant growth hormones, siderophores, and aminocyclopropane-1-carboxylate (ACC) deaminase. We conclude that *A. thaliana* can acquire significant benefits from the recruitment of streptomycetes to their root microbiome, which they likely attract through the deposition of root exudates and dead root material into the bulk soil. Additionally, mining plant-streptomycete interactions may yield novel biocontrol and plant growth-promoting agents that could be developed for future applications in agriculture.

## RESULTS

### Isolation and genome analysis of *A. thaliana* root-associated *Streptomyces* strains.

To culture bacteria from *A. thaliana* roots, the roots of 4-week-old plants growing in Levington’s seed and modular compost were washed and sonicated in sterile buffer (as described in reference 12) to remove soil particles. This process removed all bacteria apart from those that were tightly bound to the plant root surface (the rhizoplane) or were living within the plant root tissue as endophytes (12). The roots were then crushed in sterile 10% glycerol, and serial dilutions were plated onto soya flour mannitol, starch casein, and minimal salts agar. Colonies resembling streptomycetes were purified by restreaking and were then identified by colony PCR and 16S rRNA gene amplicon sequencing using the universal primers PRM341F and MPRK806R (Tables 1 and 2). Based on 16S rRNA sequencing, five phylogenetically distinct strains (L2, M2, M3, N1, and N2) were then selected for genome sequencing. We used the PacBio RSII platform (as described in reference 25) to generate high-quality genome sequences for the five isolates, in addition to three strains of Streptomyces lydicus, which are known plant endophytes (26, 27). One of these strains was isolated from the horticultural product Actinovate, and the other two came from the ATCC culture collection (Table 2). All eight linear genomes were within the size range typical for this genus and did not show any significant reductions compared to the genomes of other sequenced *Streptomyces* species (Table 1). The genomes of the *A. thaliana*-associated strains L2, M2, M3, N1, and N2 were uploaded to the automated multilocus species tree (28) for phylogenetic classification. The highest average nucleotide identity (ANI) values of strains L2, M2, and M3 to strains in the database were 88.3%, 94.7%, and 91.1%, respectively; these were below the 95% threshold that is generally used to assign strains to a known species (29, 30), and so they could be new *Streptomyces* species. Strain N1 has a 98.7% ANI to Streptomyces albidoflavus, and strain N2 has a 97.6% ANI to Streptomyces griseofuscus, suggesting they belong to these clades (Table 1).

**TABLE 1:** Genome features of root-associated *Streptomyces* strains sequenced for this study^*a*^

Strain	Accession no.	Genome size (bp)	No. of ORFs^*b*^	No. of BGCs^*c*^	Closest relative (ANI [%])^*d*^
L2	QBDT00000000	8,073,926	7,079	29	Streptomyces bungoensis (88.3)
M2	CP028834	8,718,751	8,026	22	*Streptomyces* sp. HBG00200 (94.7)
M3	QANR00000000	8,304,843	7,561	25	Streptomyces pratensis (91.1)
N1	QBDS00000000	7,207,104	6,239	21	Streptomyces albidoflavus (98.7)
N2	CP028719	8,428,700	7,401	34	Streptomyces griseofuscus (97.6)
Actinovate	RDTC00000000	9,139,876	7,989	33	*S. lydicus*
ATCC 25470	RDTD00000000	7,935,716	7,084	25	*S. lydicus*
ATCC 31975	RDTE00000000	9,244,118	8,128	31	*S. lydicus*

a Genomes were sequenced using the PacBio RSII platform.

b ORF, open reading frame.

c BGC, biosynthetic gene cluster, predicted using AntiSMASH 5.0 (38).

d Determined using AutoMLST (28).

**TABLE 2:** Strains, primers, and plasmid used in experiments

Species, strain, primer, or plasmid name	Description or sequence (5′→3′)	Origin	Genome accession no. or reference
Species or strains			
*Streptomyces* L2	Wild type	*A. thaliana* root microbiome, this study	QBDT00000000
*Streptomyces* M2	Wild type	*A. thaliana* root microbiome, this study	CP028834
*Streptomyces* M3	Wild type	*A. thaliana* root microbiome, this study	QANR00000000
*Streptomyces* N1	Wild type	*A. thaliana* root microbiome, this study	QBDS00000000
*Streptomyces* N2	Wild type	*A. thaliana* root microbiome, this study	CP028719
Streptomyces lydicus ATCC 25470	Wild type	American Type Culture Collection	RDTD00000000
Streptomyces lydicus ATCC 31975	Wild type	American Type Culture Collection	RDTE00000000
Streptomyces lydicus Actinovate	Wild type	Isolated from Actinovate by Elaine Patrick, UEA	RDTC00000000
Bacillus subtilis	Wild type, strain 168	Gift from Nicola Stanley-Wall, University of Dundee	NA^*a*^
Methicillin-resistant Staphylococcus aureus	Clinical isolate	Norfolk and Norwich University Hospital (UK)	NA
Escherichia coli K-12	Wild type	Lab stock, UEA	NA
Pseudomonas syringae DC3000	Wild type	John Innes Centre, Norwich, UK	NA
Candida albicans	Clinical isolate	Gift from Neil Gow, University of Exeter	NA
Lomentospora prolificans	Environmental isolate	American Type Culture Collection	NA
Gaeumannomyces graminis var. *tritici*	Environmental isolate	John Innes Centre, Norwich, UK	NA
Arabidopsis thaliana Col-0	Wild type, ecotype Col-0	Lab stock, UEA	NA
Triticum aestivum var. Paragon	Wild type, var. Paragon	John Innes Centre, Norwich, UK	NA
Primers			
PRK341F	CCTACGGGAGGCAGCAG		71
MPRK806R	GGACTACHVGGGTWTCTAAT		
Plasmid			
pIJ8660	ermEp* driving constitutive production of codon-optimized eGFP		66

a NA, not available.

### *Streptomyces* bacteria colonize *A. thaliana* roots.

**FIG 1:**
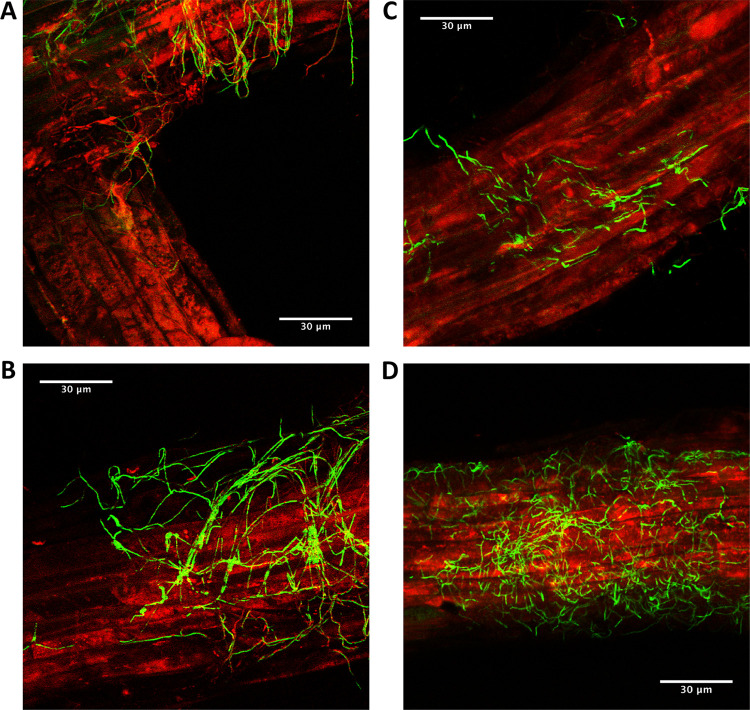
Confocal laser scanning microscopy images of *A. thaliana* rhizoplane colonization by eGFP-tagged *Streptomyces* strains 3 days after inoculation. (A and B) *A. thaliana* roots (red) colonized by eGFP-tagged Streptomyces coelicolor M145 (green), which is a known root endophyte and was used as a control (67). (C and D) *A. thaliana* roots (red) colonized by eGFP-tagged *Streptomyces* strain M3 which was isolated in this study (green).

To investigate whether the sequenced *Streptomyces* strains can promote plant growth and fitness, we established root infection assays in which seeds were coated with a suspension of pregerminated *Streptomyces* spores. Tagging the strains with enhanced green fluorescent protein (eGFP) and the apramycin resistance (*aac*) gene allowed visual confirmation of root infection using confocal microscopy (Fig. 1) and selective reisolation of the strains on agar plates containing apramycin, confirming that they were able to recolonize plant roots.

### *Streptomyces* strains M2, M3, and L2 have growth-promoting effects in *A. thaliana*.

**FIG 2:**
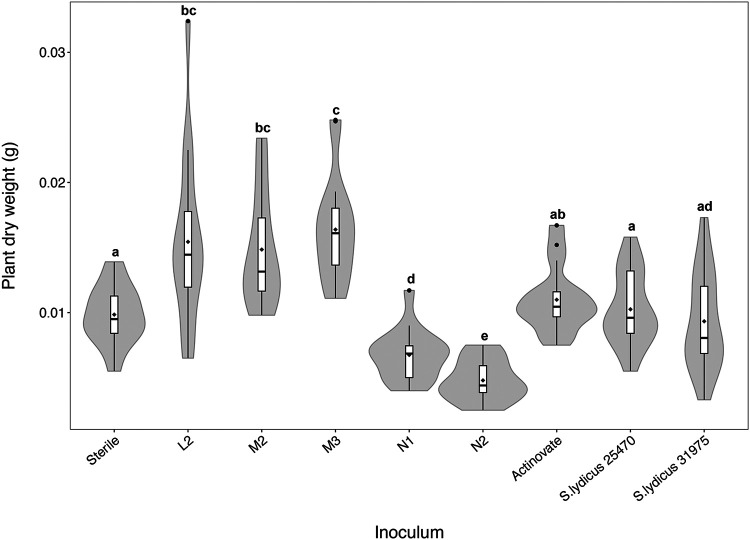
Violin plots showing the biomass of Arabidopsis thaliana plants grown on agar plates following inoculation with sequenced *Streptomyces* isolates. Biomass (dry weight in grams) was measured 16 days after inoculation. Sterile plants were grown as a control. *N* = 16 plants per treatment. Box plots show the locations of the medians and quartiles, with whiskers reaching to 1.5 times the interquartile range. ♦, mean values. The width of the outer shaded area illustrates the proportion of the data located there (the kernel probability density). Groups labeled with different lowercase letters have a significantly different plant biomass (*P* < 0.05 in Tukey’s HSD tests).

Next, we wanted to determine if any of the *Streptomyces* strains isolated from *A. thaliana* roots can enhance plant growth, and so we established root infection assays on agar plates, whereby *Streptomyces* spores were inoculated directly onto the roots of young *A. thaliana* seedlings. We tested all the genome-sequenced strains from this study and found that the inoculation of different strains had a significant effect on the dry weights of plants grown on agar compared to that of a sterile control (Fig. 2) (F_[8,135]_ = 27.63, *P* < 0.001 in an analysis of variance [ANOVA] test). Strains L2, M2, and M3 significantly increased *A. thaliana* dry biomass under these conditions compared to that of sterile control plants (Fig. 2) (*P* < 0.05 in Tukey’s honestly significant difference [HSD] tests). In comparison, the three strains of *S. lydicus* had no effect on *A. thaliana* plant biomass *in vitro* despite this species being previously noted to have plant growth-promoting effects (26). *Streptomyces* strains N1 and N2 significantly reduced the growth of *A. thaliana in vitro* relative to that of control plants (Fig. 2) (*P* < 0.05 in Tukey’s HSD).

**FIG 3:**
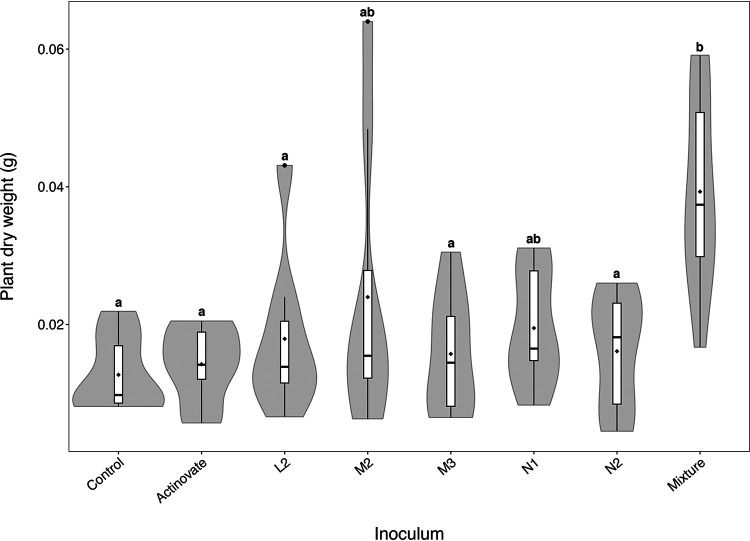
Violin plots demonstrating the total dry weight of *A. thaliana* plants grown in Levington’s F2 compost from seeds inoculated with spores of Actinovate, L2, M2, M3, N1, and N2 or a mixture of L2, M2, and M3 *Streptomyces* spores. Dry weight is shown in grams. Sterile seeds were grown as a control. *N* = 8 replicate plants per treatment. Box plots show the locations of the medians and quartiles, with whiskers reaching to 1.5 times the interquartile range. ♦, mean values. The width of the outer shaded area illustrates the proportion of data located there (the kernel probability density). Groups labeled with different lowercase letters differ significantly in plant biomass (*P* < 0.05 in Tukey’s HSD tests).

To test whether each of the new isolates could promote plant growth in soil, spores of each strain were applied individually to *A. thaliana* seeds planted in Levington’s F2 seed and modular compost. Since it is known that some strains can work synergistically to promote plant growth (31–33), a mixture of spores of L2, M2, and M3 (the strains that promoted plant growth *in vitro*) was also added to seeds. Strain inoculation had a significant influence on the dry weight of plants grown in compost (ANOVA test on log-transformed dry weight: F_[7,58]_ = 3.9358, *P* = 0.001). However, interestingly, none of the strains had an effect on plant growth when applied individually (Fig. 3) (*P* > 0.05 in Tukey HSD test), but the application of a spore mixture of L2, M2, and M3 significantly increased plant dry weight from an average of 12.69 ± 1.94 mg (mean ± standard error [SE]) for control plants to 39.29 ± 4.39 mg (Fig. 3).

### Characterization of plant growth-promoting traits.

KEGG pathway analysis revealed that the genomes of all our sequenced *Streptomyces* strains possess genes encoding proteins involved in the biosynthesis of IAA, which can contribute to shoot and root growth (see Table S2 in the supplemental material). For example, all strains have genes encoding key proteins involved in the indole-3-acetamide (IAM) pathway, whereby tryptophan is converted to IAM via a tryptophan 2-monooxygenase enzyme (KEGG reaction R00679). IAM is then further converted to IAA through the action of an amidase enzyme (KEGG reaction R03096). Several strains also possessed genes encoding enzymes involved in the tryptamine (TAM) pathway. *In vitro* colorimetric assays using Salkowski reagent (as described in reference 34) qualitatively confirmed the ability of all strains to make IAA (see Fig. S1). In addition to IAA, the genomes of all the *Streptomyces* isolates possess up to two copies of genes encoding the enzyme aminocyclopropane-1-carboxylate (ACC) deaminase. This cleaves ACC, which is the direct precursor for the plant phytohormone ethylene, into ammonia and 2-oxobutanoate (KEGG reaction R00997) (see Table S1). Bacteria can use the ammonium released in this reaction as a nitrogen source, and all the isolates are capable of utilizing ACC as a sole nitrogen source in minimal medium (see Fig. S2). There is evidence that the activity of the ACC deaminase enzyme can reduce plant damage and early-onset senescence caused by excessive ethylene production under prolonged periods of plant stress by removing the substrate for ethylene biosynthesis (35, 36).

### Bioactivity of root-associated *Streptomyces* isolates.

*Streptomyces* bacteria are well known for their ability to produce a wide range of specialized metabolites which can have bioactivity against bacteria, fungi, viruses, nematodes, insects, and plants (1, 3, 37). We reasoned that our strains likely make antimicrobial natural products and that strains N1 and N2 may encode herbicidal compounds given that they have an adverse effect on *A. thaliana* growth *in vitro* (Fig. 2). All eight sequenced genomes were submitted to the bacterial antiSMASH 5.0 portal (38), which can predict BGCs for major types of specialized metabolites. This identified between 21 and 34 putative specialized metabolite BGCs in the sequenced genomes, which is within the typical range for this genus (see Table S2). This includes BGCs predicted to encode polyketide synthases (PKS), nonribosomal peptide synthases (NRPS), ribosomally encoded posttranslationally modified peptides (RiPPs), and terpenes (Table S2). All five strains harbor multiple siderophore BGCs, which are molecules that chelate metal ions, such as iron, generating soluble complexes that can be taken up by plant roots and contribute to plant growth (39). Siderophores also help microorganisms to compete in the soil, rhizosphere, and endosphere, with the added benefit that this may act to exclude plant pathogens that are also competing for iron (40).

To test whether antimicrobial compounds could be produced *in vitro*, sequenced strains were tested for their ability to inhibit a range of bacterial and fungal pathogens, including the bacterial plant pathogen Pseudomonas syringae and the fungus *G. graminis* var. *tritici*, which is the causative agent of wheat take-all, one of the most economically damaging diseases of wheat worldwide (19). All eight strains exhibited antibacterial activity, and N2 inhibited all of the bacterial strains that were tested: Bacillus subtilis, methicillin-resistant Staphylococcus aureus (MRSA), Escherichia coli, and Pseudomonas syringae (see Fig. S3; Table S3). The N2 genome harbors a rich repertoire of BGCs, including several putative antibacterial BGCs predicted to encode the proteins responsible for the biosynthesis of albusnodin, albaflavenone, diisonitrile antibiotic SF2768-like antibiotics, and the antiproliferative actinomycin D, which is used clinically as an anticancer therapeutic, in addition to a possible analogue of the proteasome inhibitor cinnabaramide (Table S2). The actinomycins exhibit broad-spectrum bioactivity by binding to DNA and inhibiting transcription; thus, it could result in the inhibition of all the pathogenic indicator strains (41). M2 is interesting because it inhibits Pseudomonas syringae but none of the other bacteria that were tested (Table S3). It harbors a relatively modest number of BGCs, with one for a putative aquamycin-type antibiotic (Table S2). However, this effect may be due to a siderophore, because an antibacterial compound that inhibits *Pseudomonas* would also be expected to inhibit B. subtilis and E. coli (Table S3). L2 harbors clusters for the biosynthesis of albaflavenone and thioviridamide-like molecules (Table S2) and inhibits B. subtilis *in vitro* (see Table S4). M3 inhibits B. subtilis and P. syringae (Table S3) and harbors a type 3 PKS for the biosynthesis of alkylresorcinol-type phenolic lipids (Table S3), which may have antibacterial activity. Finally, N1 inhibits B. subtilis (Table S3) and harbors several BGCs encoding molecules with potential antibacterial activity, including a putative polycyclic tetramate macrolactam (PTM) antibiotic, which is widely distributed but often cryptic (42), and a desotamide-like antibiotic (Table S2). It should be noted that up to 90% of specialized metabolite BGCs are cryptic (i.e., not expressed under laboratory conditions) in *Streptomyces* species, largely because we do not understand the environmental or host-derived signals that activate their expression (3). In addition, there are many known specialized metabolites for which the BGCs have not yet been identified, and so it is likely that at least some of the known BGCs in these genomes are not expressed under the growth conditions used in this study and/or that these strains encode antimicrobials whose BGCs cannot be predicted by antiSMASH 5.0.

The strains L2, M2, and M3 did not exhibit antifungal activity against Candida albicans, Lomentospora prolificans, or *G. graminis* var. *tritici in vitro* (Table S3), although it is possible that these strains may harbor cryptic antifungal compounds that are not expressed under the conditions used in experiments. For example, M3 harbors a clavam-like cluster, which has a similar structure to those that encode the antifungal compound clavamycin (Table S2). In comparison, strains N1 and N2 both have type 1 (T1) PKS gene clusters, which are predicted to encode the biosynthesis of known polyene antifungal compounds (Table S2). The N1 T1PKS gene cluster is predicted to encode candicidin, and the N2 T1PKS gene cluster is predicted to encode filipin-type antifungals (Table S3). Both of these metabolites inhibit fungal growth by binding to sterols in their cell walls, and it is known that filipins can also bind to phytosterols in plant cell membranes and reduce the growth of plant roots at high concentrations (43–45). Thus, it is possible that N1 and N2 are making polyenes *in planta* and this is what caused the reduction in growth observed during *in vitro* inoculation experiments (Fig. 2). These strains did not influence plant growth in compost (Fig. 3), suggesting that the effect was diluted in a nonsterile system or that the polyenes were not expressed under these conditions. Despite the presence of antifungal-like clusters in the other *A. thaliana* isolates, only N2 exhibited antifungal activity *in vitro* (Table S3). In fact, N2 demonstrated potent and broad-spectrum antifungal activity as it inhibited all three test strains; these were the human-pathogenic fungal species Candida albicans and multidrug-resistant *Lomentospora prolificans* as well as the plant-pathogenic take-all fungus Gaeumannomyces graminis var *tritici* (Table S3).

**FIG 4:**
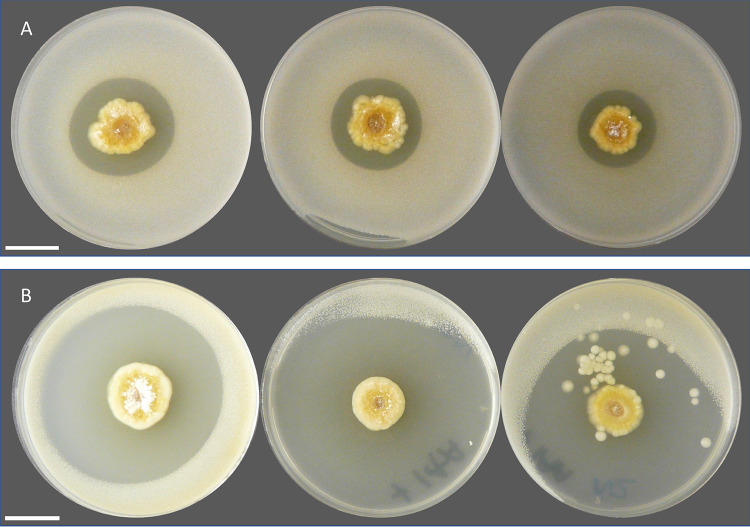
(A) Three biological replicates of strain N2 (center) growing on minimal medium agar that has been overlaid with soft LB agar inoculated with Candida albicans. (B) Same as for panel A but the minimal medium agar contains 0.1 mg ml^−1^ of indole-3-acetic acid. This experiment was repeated 4 times (each time with 3 replicates), with consistent results. Bars, 2 cm.

The production of antifungal compounds likely gives N2 an advantage in the rhizosphere and, consistent with this, N2 antifungal activity against C. albicans was shown to increase 2-fold *in vitro* in the presence of indole-3-acetic acid (IAA), as judged by the size of the inhibition zone (Fig. 4). IAA is the precursor to the plant phytohormone auxin, which regulates processes involved in growth and development (46). It is also made by many microbial species in the rhizosphere, including the bacterial strains that were isolated in this study (Table 2; Fig. S1), and has been noted previously for its role in both intra- and interkingdom signaling (46–48). Thus, N2 may be driven to produce antifungals in close proximity to plant roots when it is in competition with other microbes.

### Purification and identification of antifungal compounds from *Streptomyces* strain N2.

**FIG 5:**
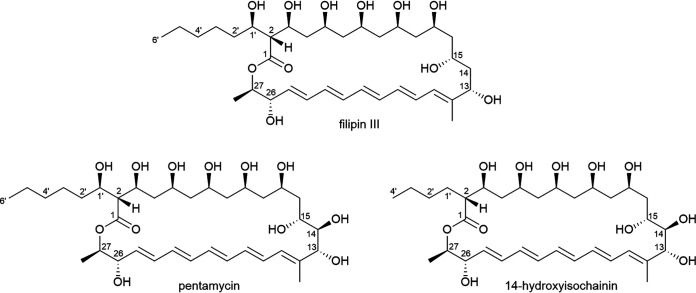
Structures of filipin III, pentamycin, and 14-hydroxyisochainin.

To extract the molecules with antifungal activity, strain N2 was plated on soya flour-mannitol (SFM) agar (4 liters, 120 plates) and grown for 8 days at 30°C before extraction with ethyl acetate. The crude extract had an intense orange color, and high-performance liquid chromatography (HPLC) analysis identified components with UV characteristics typical of polyene metabolites. Comparison to an authentic commercial sample of the filipin complex of Streptomyces filipinensis (Sigma-Aldrich) confirmed the presence of several filipin-related molecules, although the two major components eluted earlier than these molecules (see Fig. S4). The two major components eluted very closely together and had *m/z* values consistent with the previously reported molecules pentamycin (also known as fungichromin) and 14-hydroxyisochainin (49–51). These filipin-like compounds (Fig. 5) can all be assigned to the type 1 PKS BGC in region 1 (Table S2; Fig. S17) (50, 52, 53). Despite a challenging elution profile, sufficient separation was accomplished by applying multiple purification steps, and the isolated material enabled structural confirmation by two-dimensional (2D) nuclear magnetic resonance (NMR) (Fig. S5 to S16, Tables S4 to S6) accompanied by comparison to published data (49–51) and bioinformatics analysis of the associated PKS-encoding genes in strain N2 (52–54). The extract also contained a mixture of components with an absorption maximum at 444 nm, and this mixture was red after separation from the polyene fraction. Liquid chromatography-mass spectrometry (LC-MS) analysis indicated that these components were consistent with the known actinomycin congeners D, X_2_, and X_0β_ (Fig. S4). These are most likely encoded by BGC5 in the N2 genome (Table S2) (55).

Pentamycin is active against Candida albicans and Trichomonas vaginalis and is used for the treatment of vaginal candidiasis, trichomoniasis, and some mixed infections (56–58). It is identical in structure to filipin III apart from the presence of an additional secondary hydroxyl group at C-14 (Fig. 5), which is added by a cytochrome P450 monooxygenase encoded by a gene located directly upstream of the first PKS gene (A1) in the pentamycin BGC as reported recently (50); the same BGC architecture is observed in *Streptomyces* strain N2 (see Fig. S17). 14-Hydroxyisochainin shares the same polyene core structure as pentamycin but carries an altered side chain lacking two carbon atoms and is indicative of different length extender units being utilized by the final module of the type I PKS during biosynthesis (Fig. 5). This observation is unexpected, as the coproduction of pentamycin and 14-hydroxyisochainin has only been observed as a result of precursor-directed biosynthesis (51). Thus, this example of coproduction by *Streptomyces* strain N2 appears to be novel. Disc diffusion bioassays confirmed that pentamycin and 14-hydroxyisochainin inhibit the growth of C. albicans (see Fig. S18), but only 14-hydroxyisochainin was able to inhibit *G. graminis* var. *tritici* (see Fig. S19), suggesting that 14-hydroxyisochainin is responsible for the antifungal activity of N2 against the take-all fungus, potentially in combination with lower quantities of other products of the filipin-like BGC. The complex consisting of actinomycin D, X_2_, and X_0β_, which were copurified from N2 extracts (Fig. S4), did not have antifungal activity, suggesting they are not responsible for N2 antifungal bioactivity *in vitro* (Fig. S18). Neither pentamycin, 14-hydroxyisochainin, nor the actinomycin complex inhibits the growth of E. coli K-12 (Fig. S18), suggesting that a previously undescribed compound is responsible for the observed inhibition of E. coli and P. syringae by N2 *in vitro* (Fig. S3; Table S3).

### *Streptomyces* strain N2 protects wheat against take-all.

**FIG 6:**
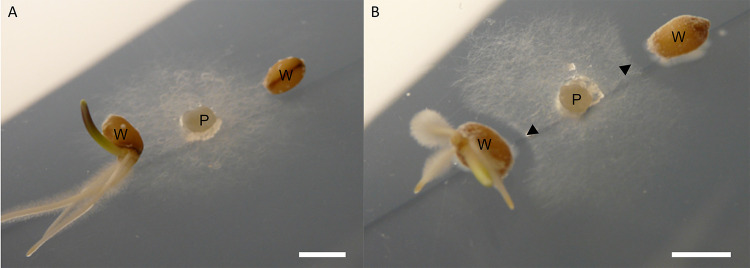
Inhibition of *G. graminis* var. *tritici* in wheat seedlings. Germinating wheat seeds (W) are either sterile (A) or inoculated with a spore preparation of *Streptomyces* isolate N2 (B), growing next to a plug of *G. graminis* var. *tritici*, the take-all fungus (P). *G. graminis* is prevented from growing toward inoculated seeds, as demonstrated by the zone of inhibition (marked with arrowheads). Bars, 5 mm.

To test whether strain N2 has the potential to protect wheat plants against *G. graminis* var. *tritici*, we inoculated surface-sterilized wheat seeds (Triticum aestivum var. Paragon) with N2 spores or left them sterile as a control. These seeds were germinated next to a central plug of take-all fungus on potato glucose agar (PGA) plates. On the control plates, *G. graminis* grew outwards across the agar plate and over the sterile wheat seedlings, whereas the seeds that had been inoculated with N2 spores were resistant to *G. graminis* var. *tritici*, as indicated by a zone of inhibition around the germinating wheat seeds (Fig. 6). The most parsimonious explanation is that the N2 spores have germinated and are producing 14-hydroxyisochainin and possibly other filipins that inhibit the take-all fungus.

**FIG 7:**
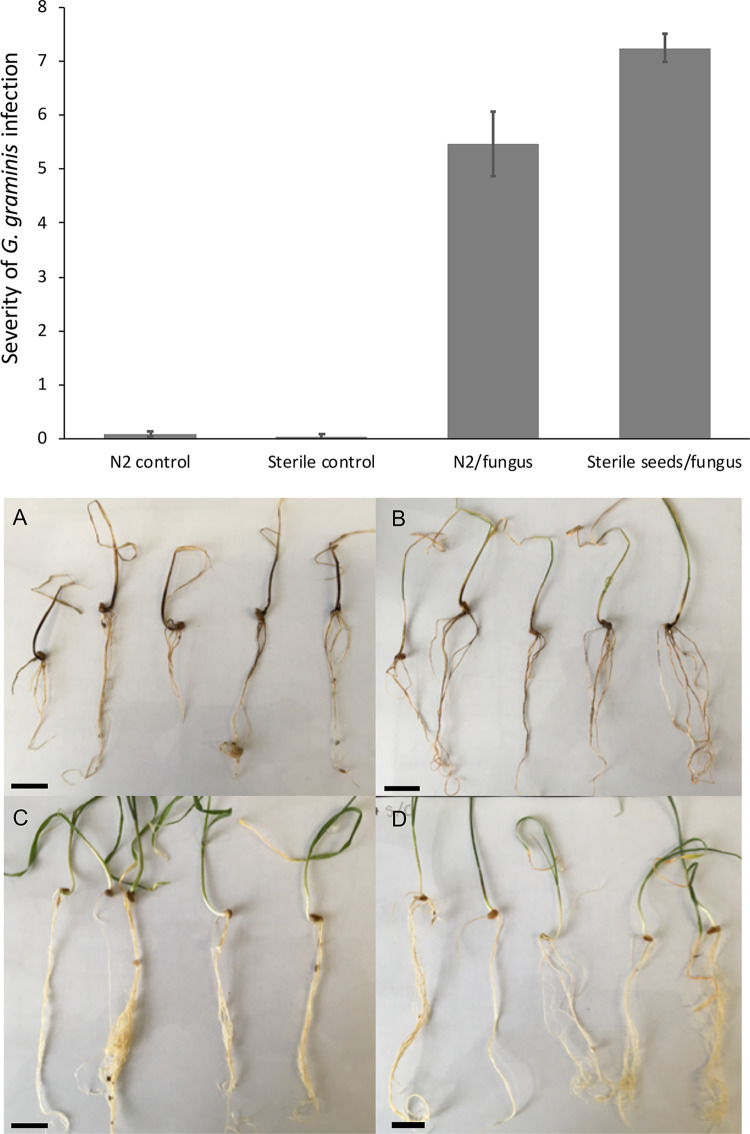
(Top) The effect of *Streptomyces* strain N2 on wheat plant infection severity by *G. graminis* var. *tritici*. Infections were scored after 3 weeks of growth. N2 control, seeds coated in N2 spores/no *G. graminis*; sterile control, sterile seeds/no *G. graminis*; N2/fungus, seeds coated in N2 spores grown in the presence of *G. graminis*; sterile seeds/fungus, sterile seeds grown in the presence of *G. graminis. N* = 25 plants per treatment group; error bars represent standard errors. Wheat plants were grown from sterile seeds in the presence of *G. graminis* var. *tritici* (A), from seeds inoculated with N2 spores, in the presence of *G. graminis* var. *tritici* (B), from sterile seeds, no *G. graminis* var. *tritici* (C), or from seeds coated with N2 spores, no *G. graminis* var. *tritici* (D). Bars, 2 cm.

To further test the potential of the *Streptomyces* strain N2 to act as a biocontrol strain against take-all *in vivo*, wheat seeds were soaked in N2 spores, allowed to dry, and then grown in sterile vermiculite containing *G. graminis* var. *tritici* mycelia. After 3 weeks of growth at 25°C, take-all infection severity was scored on a scale of 0 to 8 using an infection scoring system as follows: 0, no infection; 1, maximum of one lesion per root; 2, more than one lesion per root; 3, many small and at least one large lesion per root; 4, many large lesions per root; 5, roots completely brown; 6, roots completely brown plus infection in stem; 7, roots completely brown, infection in stem, and wilted yellow leaves; 8, entire plant brown and wilted (see Fig. S20). The wheat plants that had germinated and grown in the absence of take-all (with or without N2 spores) were healthy and infection free (Fig. 7). However, those seeds that had grown from uninoculated seeds in the presence of the take-all fungus showed extensive and severe levels of take-all disease, with an average infection score of 7.24 ± 0.26 (standard error [SE]) (Fig. 7). Most of the plants in this treatment group exhibited infected roots, stems, and leaves, which all appeared senescent and brown (Fig. 7). However, there was a significant effect of plant treatment on infection score (Kruskal-Wallis test, H_DF=3_ = 83.41, *P* < 0.001). Plants that had grown from seeds coated in N2 spores (*N* = 25) demonstrated a small, but significant, decrease in average infection severity, to 5.47 ± 0.59, compared to that for plants grown from uninoculated seeds in the presence of take-all (Fig. 7) (Dunn’s test between inoculated and sterile wheat grown with *G. graminis*, *P* = 0.023). As plants were grown in a sterile system in this experiment, it is likely that the streptomycete experienced low levels of nutrient availability compared to that in a natural soil environment. We hypothesize that greater levels of competition and nutrients may fuel greater levels of antibiotic production by N2; thus, the strain could offer greater levels of protection against host infection in a more natural soil-plant system.

## DISCUSSION

*Streptomyces* species have traditionally been described as free-living soil bacteria, but given that most bare soils are rapidly colonized by vegetation, it is perhaps not surprising that they are also effective at colonizing the rhizosphere and endosphere of plants (10). It has even been suggested that their filamentous growth may have evolved to facilitate plant root colonization, since this trait evolved 50 million years after plants first colonized land, around 450 million years ago (mya) (2, 10). Certainly, their ability to sporulate provides a useful mechanism for vertical transmission across plant generations via the soil. In this study, we followed up reports that *Streptomyces* bacteria are enriched in the *A. thaliana* rhizosphere and specifically recruited by plant-produced compounds such as the plant hormones salicylate and jasmonate (12–17). We aimed to isolate and characterize plant-associated streptomycetes from *A. thaliana* and test whether they can be beneficial to their plant host. We generated high-quality genome sequences for five *A. thaliana* root-associated strains and three strains of the known endophyte *S. lydicus*. We found that three of the five root-associated strains significantly increased the biomass of *A. thaliana* plants when they were applied to seeds or roots, both *in vitro* and when applied in combination in soil. Two others, N1 and N2, significantly decreased the biomass of *A. thaliana in vitro*, most likely because they make polyenes that bind to sterols, including the phytosterols found in plant cell walls (43–45), which probably had a negative effect on the plant in a sterile system. However, this effect was removed in a compost system, with neither strain influencing plant growth. Our work suggests, therefore, that while *Streptomyces* species are consistently associated with *A. thaliana* roots and enriched in the root microbiome compared to that in the surrounding bulk soil, not all strains that are competitive in the rhizosphere and endosphere necessarily have a beneficial effect on host fitness. This is important from an ecological and applied perspective: the former because it helps us to better understand the microbial and host factors influencing plant microbiome assembly, and the latter because tipping the balance in the plant’s favor, for example, by applying beneficial species as seed coatings or soil additives that can competitively colonize roots, could improve crop yields (19, 59, 60). Such strains could be used as biological growth promoters to replace the use of harmful pesticides and fertilizers, which have negative effects on the wider ecosystem and also contribute to climate change (10, 11, 19, 59, 60). As a proof of this concept, we took strain N2, which makes the polyene antifungals, including pentamycin, 14-hydroxyisochainin, and filipin III, and coated seeds of spring bread wheat with its spores. N2 was able to protect germinating wheat seedlings against the take-all fungus *in vitro* and significantly reduce take-all disease progression in wheat plants grown in sterile vermiculite. Although we do not have an assay for take-all disease in soil, the protective effect may be even greater, because nutrients in the form of organic matter could provide a more beneficial growing environment for the *Streptomyces* strain than sterile vermiculite (61) and the presence of a greater diversity of microbes may fuel antimicrobial production by this strain as a result of interference competition (62). It is also intriguing that IAA increased the antifungal activity of strain N2, since it provides compelling evidence that environmental signals can alter the expression of secondary metabolites and, probably as an extension, also influence interspecies competition (46, 48). Although there is much future work to do to understand this phenomenon, our results provide a system to begin to understand how secondary metabolites are regulated and used by microbes in nature and may also provide new tools for activating the 90% of BGCs that are silent in these bacteria. Plant-associated *Streptomyces* strains may yet provide us with a new generation of antimicrobials for the clinic and might also be harnessed to improve our food security. Understanding the ecology of these bacteria and their associated natural products will be crucial if we are to achieve these goals.

## MATERIALS AND METHODS

### Isolation of root-associated *Streptomyces* strains.

Recipes for buffers and media are listed in Table S7 in the supplemental material. Strains, primers, and plasmids are listed in Table 2. Wild-type *A. thaliana* Col-0 seeds were sterilized by washing in 70% (vol/vol) ethanol for 2 min, 20% (vol/vol) sodium hypochlorite for 2 min, and then five times in sterile water. Individual seeds were sown into pots of sieved Levington’s F2 seed and modular compost, placed at 4°C for 24 h, and then grown for 4 weeks under a 12-h photoperiod (12 h light/12 h dark) at 22°C. Plants were taken aseptically from pots, and their roots were tapped firmly to remove as much adhering soil as possible. Root material was placed into sterile Silwet L-77-amended phosphate-buffered saline (PBS-S) for 30 min on a shaking platform and then transferred into fresh PBS-S and washed for 30 min. Any remaining soil particles were removed with sterile tweezers. Cleaned roots were then transferred to 25 ml of fresh PBS-S and placed in a sonicating water bath for 20 min to remove any residual material still attached to the root surface; this was to ensure that any remaining bacteria either were present in the endophytic compartment or were very firmly attached to the root surface (“the rhizoplane”) (12). The roots were crushed in sterile 10% (vol/vol) glycerol, and serial dilutions were spread onto either soya flour-mannitol (SFM) agar, starch casein agar, or minimal salts medium agar containing sodium citrate. Plates were incubated at 30°C for up to 14 days. Colonies resembling streptomycetes were restreaked on SFM agar and identified by 16S rRNA gene PCR amplification and sequencing with the universal primers PRK341F and MPRK806R. Sequencing was carried out by Eurofins Genomics, Germany. Three strains of Streptomyces lydicus, which are known to associate with plant roots, were also used in experiments; *S. lydicus* WYEC108 was isolated from the commercial biocontrol product Actinovate, and two more *S. lydicus* strains (ATCC 25470 and ATCC 31975) were obtained from the American Type Culture Collection. *Streptomyces* strains were maintained on SFM agar (N1, N2, M2, and M3), maltose-yeast extract-malt extract (MYM) agar with trace elements (L2), or ISP2 agar (*S. lydicus* strains). Strains were spore stocked as described previously (63).

### Genome sequencing and analysis.

High-quality genome sequences were obtained for *Streptomyces* strains N1, N2, M2, M3, and L2 as well as the three known strains of Streptomyces lydicus using PacBio RSII sequencing technology at the Earlham Institute, Norwich, UK, as described previously (25). The automated multilocus species tree (autoMLST) server (28) was used to phylogenetically classify the *Streptomyces* strains L2, M2, M3, N1, and N2. BGCs were predicted using antiSMASH 5.0 (38), and genomes were annotated using RAST (64). Amino acid sequences were uploaded to the KEGG Automatic Annotation Server (KAAS) for functional annotation of genes and metabolic pathway mapping (65).

### Generating eGFP-labeled *Streptomyces* strains.

Plasmid pIJ8660 containing a codon-optimized eGFP gene under the control of the constitutive ermE* promoter and the *aac* apramycin resistance marker (66) was conjugated into *Streptomyces* strains (63). Exconjugants were selected and maintained on SFM agar plates (Table S7) containing 50 μg ml^−1^ apramycin. For confocal microscopy, *A. thaliana* Col-0 seeds were germinated on Murashige and Skoog (MSk) agar (Table S7) and grown vertically at 22°C for 9 days under a 12-h photoperiod. Seedlings were then transferred to MSk (1.5% agar, 0% sucrose) (Table S7) and allowed to equilibrate for 24 h before being inoculated with 1 μl of spore suspension (10^6^ spores ml^−1^) of the eGFP-tagged M3 or Streptomyces coelicolor M145 strains. As a known colonizer of plant roots, S. coelicolor was used as a control (67). Inoculated seedlings were then left to grow for 3 days before being washed in a 20% (vol/vol) solution of glycerol containing 1 μg ml^−1^ 276 SynaptoRed for 10 min. A 20-mm section of root (taken from the base of the petiole) was then mounted onto a slide with 100 μl of the SynaptoRed-glycerol solution. Samples were imaged using a Zeiss LSM510 META laser-scanning confocal microscope with a PlanApochromat 63× (1.4 numerical aperture [NA]) lens objective. Green fluorescent protein was excited at 488 nm, and emission was collected through a 527.5 ± 22.5-nm bandpass (bp) filter; FM4-64 was excited at 543 nm, and emission was collected through a 587.5 ± 27.5-nm bp filter.

### Plant growth promotion assays.

*A. thaliana* Col-0 seeds were sterilized and plated onto MSk medium (1% [wt/vol] sucrose and 0.8% [wt/vol] agar) (Table S7). These were then left at 4°C in the dark for 24 h before being placed, vertically, under long-day growth conditions (12 h light/12 h dark) at 22°C for 10 days. Seedlings were then transferred to square agar plates containing MSk agar (as described above) (Table S7) and allowed to equilibrate, vertically, overnight at 22°C. One microliter of *Streptomyces* spores (10^6^ ml^−1^) from each of the sequenced isolates was added to the top of the root system of each seedling and allowed to dry. Sixteen replicate seedlings were inoculated per sequenced *Streptomyces* strain; 10% (vol/vol) glycerol was added to control seedlings. Plates were grown vertically for 16 days with 12 h light/12 h dark at 22°C before plant biomass (dry weight) was measured. The biomasses of plants with different inocula were compared via ANOVA and Tukey’s honestly significant difference (HSD) tests using R 3.2.3 (68); biomass was log-transformed during analyses to ensure normality of residuals. Strains were also tested for their ability to promote *A. thaliana* growth in compost. Sterile *A. thaliana* Col-0 seeds were placed into a solution of 2× yeast extract-tryptone (YT) (Table S7) containing 10^6^ pregerminated spores ml^−1^ of each strain or no spores as a control. Seeds were incubated in the spore solution for 2 h before being transferred to pots containing sieved Levington’s F2 seed and modular compost. An additional 3 ml of pregerminated spores (or sterile 2× YT) was pipetted into the soil surrounding each seed. The strains L2, M2, and M3 were also tested for their ability to promote plant growth in combination; 10^3^ spores ml^−1^ of each strain were mixed together and pregerminated in 2× YT before being used as above. Pots were then placed at 4°C for 48 h before being grown for 6 weeks under a photoperiod of 12 h light/12 h dark. There were 8 replicate pots per treatment. After 6 weeks, the plants were removed from pots and cleaned by washing in PBS-S (Table S7) and using tweezers to remove adhering soil particles. Plants were then dried in an oven at 50°C, enabling plant dry weight to be calculated. An ANOVA test and Tukey’s HSD tests were used (as described above) to test for an effect of strain inoculation on plant dry weight. Dry weights were log-transformed prior to analysis.

### Indole-3-acetic acid production assays.

*Streptomyces* isolates were grown on cellophane membranes covering yeast-peptone-dextrose (YPD) medium supplemented with 5 mM tryptophan (Table S7). After 7 days, cellophane membranes with bacterial biomass were removed, and plates were flooded with Salkowski reagent (as described in reference 34). A red color indicates that IAA has leached into the medium.

### 1-Aminocyclopropane-1-carboxylic acid degradation assays.

To test for the use of ACC as a sole nitrogen source, *Streptomyces* strains were streaked onto Dworkin and Foster medium (69) in which 0.2% (wt/vol) NH_4_SO_4_ or 0.051% (wt/vol) ACC was added as a sole nitrogen source or with no nitrogen source as a control. Plates were incubated for 10 days at 30°C before imaging.

### Antibiotic bioassays.

Spores (4 μl of 10^6^ ml^−1^ solution) of individual *Streptomyces* isolates were pipetted onto the centers of agar plates and incubated at 30°C for 7 days before adding the pathogenic indicator strains (see Table 2). A clinical isolate of Candida albicans (gift from Neil Gow), Bacillus subtilis 168 (from Nicola Stanley-Wall), a clinical isolate of methicillin-resistant Staphylococcus aureus isolated from a patient at the Norfolk and Norwich University Hospital UK (70), an Escherichia coli K-12 lab strain, and the plant pathogen Pseudomonas syringae DC3000 (gift from Jacob Malone) were grown overnight in 10 ml lysogeny broth (LB) at 30°C, 250 rpm. These were subcultured 1 in 20 (vol/vol) for a further 4 h at 30°C before being used to inoculate 100 ml of molten LB (0.5% agar), 3 ml of which was used to overlay each agar plate containing a *Streptomyces* colony. Plates were incubated for 48 h at 30°C. Bioactivity was indicated by a clear halo around the *Streptomyces* colony. For bioassays using the fungal strain *Lomentospora prolificans* or Gaeumannomyces graminis var. *tritici* (Table 2), a plug of the fungus (grown for 14 days on potato glucose agar) was placed at the edge of the agar plate, 2 cm away from the growing streptomycete colony. Plates were incubated at 25°C for up to 14 days to assess inhibition of fungal growth. Bioassays were carried out on a range of different media, including minimal medium supplemented with indole-3-acetic acid (IAA) (see Table S7 for medium recipes).

### Purification and elucidation of filipin-like compounds from strain N2.

Spores of strain N2 were spread onto 120 plates (4 liters) of SFM agar and grown for 8 days at 30°C. The resulting agar was then sliced into small pieces and extracted with ethyl acetate (3 × 1.5 liters). An analytical sample was taken for analysis whereby the extract was filtered through gauze and the solvent removed under reduced pressure, yielding 9.2 g of crude material. This was split into two halves, and each was treated identically: after dissolving in acetone (50 ml), loose normal-phase silica gel (∼30 g; Sigma-Aldrich) was added and the solvent was removed under reduced pressure. The impregnated silica gel was dry loaded onto a Biotage SNAP Ultra cartridge (50 g, HP-Sphere normal-phase silica). The resulting sample was chromatographed using a Biotage flash chromatography system to separate the polyene fraction (338 nm) and the actinomycin(s)-containing fraction (444 nm; here referred to as the “actinomycin complex”) using the following gradient with a flow rate of 100 ml min^−1^: hold at 0% B for 1 column volume (CV); linear gradients of 0% to 50% B over 10 CVs and 50% to 100% B over 0.5 CVs; hold at 100% B for 3 CVs (mobile phase A, chloroform; mobile phase B, methanol).

The polyene-containing fractions were combined, the solvent was removed under reduced pressure, and the residues were split into two fractions. Each fraction (in 800 μl dimethyl sulfoxide [DMSO]) was loaded onto a Biotage SNAP Ultra cartridge (12 g, C_18_), and chromatography was achieved using the following gradient at a flow rate of 12 ml min^−1^: hold at 0% B for 5 CVs; linear gradients of 0% to 55% B over 1 CV, 55% to 85% B over 10 CVs, and 85% to 100% B over 2 CVs; hold at 100% B for 1 CV (mobile phase A, water; mobile phase B, methanol). The resulting fractions were analyzed using LC-MS and combined to yield three polyene samples of 53 mg, 34 mg, and 9 mg after solvent was removed. LC-MS spectra of these fractions and the original crude extract were then uploaded onto the GNPS (Global Natural Products Social Molecular Networking) platform. The largest network containing the spectra of the filipin-related compounds was manually adapted and processed in Cytoscape 3.6.1. The second fraction contained the most interesting compounds, and so only this sample was further purified by chromatography over a Synergi Fusion 4-μm C_18_ 250-mm by 10-mm column (Phenomenex) using an Agilent 1100 series HPLC system fitted with a fraction collector and eluting at a flow rate of 3.5 ml min^−1^ with the following gradient: 0 to 2 min, 45% B; 2 to 5 min, 45% to 50% B; 5 to 10 min, 50% B; 10.0 to 10.1 min, 50% to 45% B; 10.1 to 12.0 min, 45% B (mobile phase A, 0.1% formic acid in water; mobile phase B, acetonitrile). This yielded pentamycin (4.2 mg) and 14-hydroxyisochainin (2.3 mg). Both structures were assigned using 2D NMR recorded on a Bruker Avance Neo 600-MHz spectrometer equipped with a helium-cooled cryoprobe and dissolved in DMSO-*d*_6_. Absolute structures were assigned based on the identical chemical shifts displayed in comparison to published data, and additional one-dimensional (1D) experiments were carried out in CD_3_OD for 14-hydroxyisochainin in order to compare directly with the published data for this compound (Fig. S10 to S16 and Tables S5 to S8) (51).

Disc-diffusion bioassays were used to test whether purified pentamycin, 14-hydroxyisochainin, and the actinomycin complex were active against the pathogenic strains C. albicans and E. coli (Table 2). Both indicator strains were grown in 10 ml of LB broth (Table S7) at 30°C and 200 rpm overnight. Cultures were then diluted 1 in 20 (vol/vol) in 10 ml of LB and grown for a further 4 h. The 10-ml subculture was added to 50 ml of soft LB (0.5% agar), which was then poured into 10-cm^2^ plates and allowed to set. Meanwhile, 6-mm sterile filter paper discs (Whatman) were inoculated with 40 μl of each individual compound (three technical replicates of each compound were tested). Forty microliters of methanol was added to one disc per plate as a solvent control, and 40 μl of nystatin (5 mg ml^−1^) or hygromycin (50 mg ml^−1^) was used as a positive control on C. albicans or E. coli plates, respectively. Once dry, discs were placed onto plates 3 cm apart. These were then incubated at 30°C overnight. Inhibition of the indicator strain was evidenced by a zone of clearing around the disc. Purified compounds were also tested for their ability to inhibit the wheat take-all fungus *G. graminis* (Table 2). For this, discs were placed onto PGA plates (Table S7) 2 cm away from an actively growing plug of *G. graminis*. Plates were left to grow at room temperature for 5 days before imaging. Three technical replicates were run for each purified compound (pentamycin, 14-hydroxyisochainin, and actinomycin).

### Wheat seedling bioassays with *Streptomyces* strain N2.

Seeds of Triticum aestivum (var. Paragon) (Table 2) were sterilized by placing them in 70% (vol/vol) ethanol for 2 min followed by a wash in 3% (vol/vol) NaOCl for 10 min. Seeds were then rinsed five times in sterile distilled water (dH_2_O) before placing them into a solution of pregerminated spores (10^7^ spores ml^−1^) of *Streptomyces* strain N2 (Table 2). Spores were pregerminated in 2× YT (Table S7) at 50°C for 10 min. Seeds were incubated in either the spore preparation or sterile 2× YT as a control for 2 h before being allowed to dry in a petri dish under sterile conditions. Two wheat seeds were then placed on a 10-cm^2^ plate of MSk agar (1.5% [wt/vol] agar, 0% [wt/vol] sucrose) (Table S7) on either side of a plug of the *G. graminis* fungus, which was placed in the center of the agar plate. Plugs were taken from an actively growing plate of *G. graminis* on PGA. Three replicate plates each of N2-coated seeds and sterile control seeds were used in each experiment. Plates were incubated for 5 days at room temperature, after which inhibition of *G. graminis* was indicated by a zone of clearing around the wheat seeds.

A sterile vermiculite system was used to investigate the ability of *Streptomyces* strain N2 to protect older wheat seedlings against take-all infection. Twenty-five milliliters of sterile vermiculite was placed into the bottom of a 50-ml Falcon tube. Five plugs of *G. graminis* actively growing on PGA (Table S7), or uninoculated PGA plugs as a control, were placed on top of this layer before being covered with a further 10 ml of vermiculite. Five seeds of *T. aestivum* (soaked in either N2 spores or uninoculated 2× YT, as described above) were then placed on top of this vermiculite layer before the addition of a further 10 ml of vermiculite. The Falcon tubes were then sealed with parafilm and incubated at 25°C for 3 weeks under a 12-h light/12-h dark photoperiod. There were five replicate tubes, each containing five replicate seeds, of each of the following combinations: PGA plugs with N2-coated seeds (wheat-*Streptomyces* control), *G. graminis* plugs with N2-coated seeds (wheat-*Streptomyces*-fungus treatment), PGA plugs with uninoculated seeds (wheat control), and *G. graminis* plugs with uninoculated seeds (wheat-fungus treatment). After 3 weeks of incubation, plants were taken from the Falcon tubes, and adhering vermiculite was removed from the roots. Take-all infection severity was scored on a scale of 0 to 8 using an infection scoring system as follows: 0, no infection; 1, maximum of one lesion per root; 2, more than one lesion per root; 3, many small and at least one large lesion per root; 4, many large lesions per root; 5, roots completely brown; 6, roots completely brown plus infection in stem; 7, roots completely brown, infection in stem, and wilted yellow leaves; 8, entire plant brown and wilted. Differences in infection scores between treatments were analyzed in R 3.2.3 (68) using a Kruskal-Wallis test coupled with a *post hoc* Dunn’s multiple-comparison test.

### Data availability.

Genome accession numbers for the strains sequenced in this study were deposited in GenBank under accession numbers QBDT00000000, CP028834, QANR00000000, QBDS00000000, CP028719, RDTD00000000, RDTE00000000, and RDTC00000000 and are listed in Table 2.

## Supplementary Material

Supplemental file 1

Supplemental file 2
