# Supplementary material for: Streptomyces Endophytes Promote Host Health and Enhance Growth across Plant Species
Source: Appl Environ Microbiol. 2020 Aug 3;86(16):e01053-20. doi: 10.1128/AEM.01053-20 (PMC7414947; doi:10.1128/AEM.01053-20)
Supplement: Supplemental file 1 [file AEM.01053-20-s0001.pdf]

Supplementary Figures

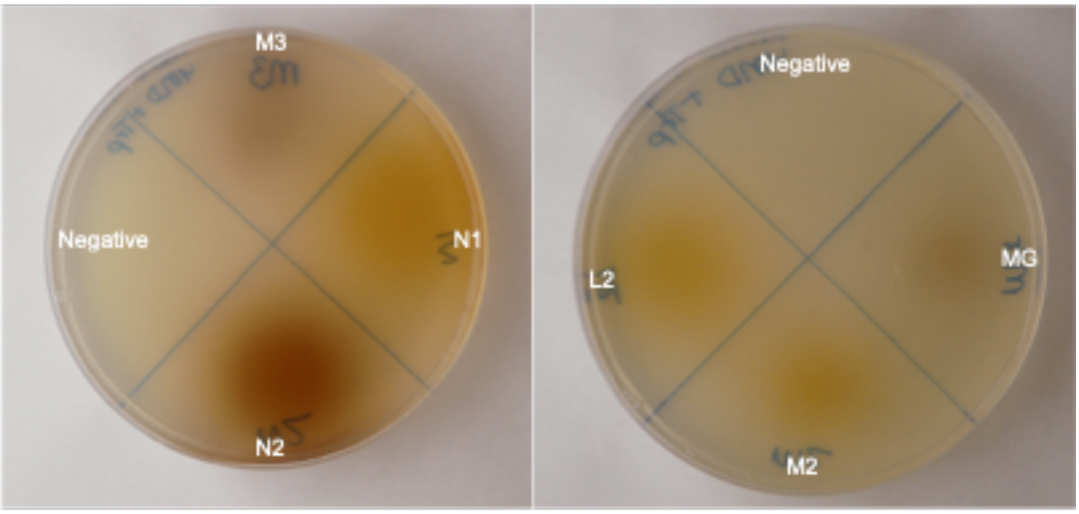

Figure S1. The *in vitro* production of IAA by *Streptomyces* isolates M3, N1, L2, N2, M2 and MG. Isolates were grown on cellophanes covering YMD agar supplemented with 5 mM tryptophan for 7 days. Cellophanes were removed and plates were flooded with Salkowski reagent. A red/pink colour indicates IAA has leached into the media. Negative = no bacteria were grown on this part of the cellophane.

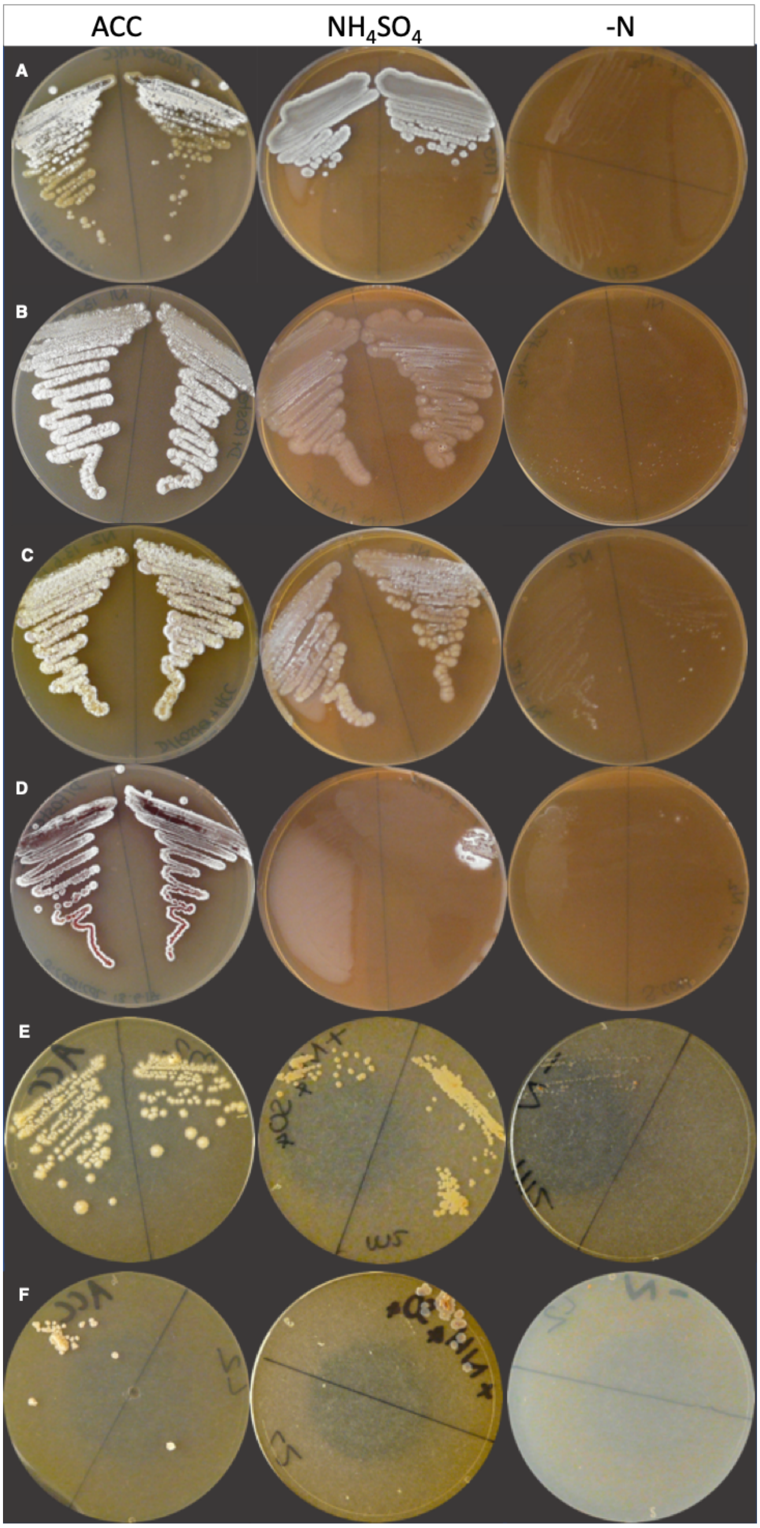

Figure S2. The use of ACC as a sole nitrogen source. *Streptomyces* strains A) M3, B) N1, C) N2, D) *Streptomyces coelicolor* M145, E) M2 and F) L2 were grown on Dworkin and Foster medium containing either 1-aminocyclopropane-1-carboxylic acid (ACC) or $NH_4(SO)_4$ as a sole nitrogen source, or no nitrogen (-N) as a control

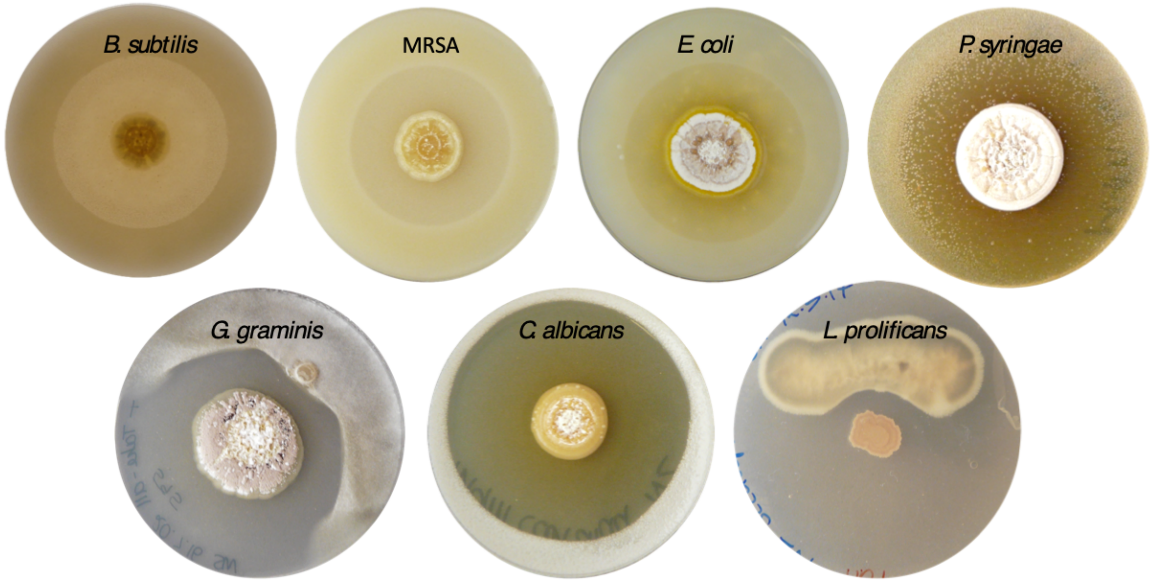

Figure S3. Bioassay plates demonstrating the ability of the *Streptomyces* isolate N2 (inoculated on the centre of the plates) to inhibit the growth of the Gram-positive bacteria *B. subtilis* and methicillin-resistant *Staphylococcus aureus* (MRSA), the Gram-negative bacteria *E. coli* and *P. syringae* as well as the fungal pathogens *Candida albicans*, *G. graminis* var. *tritici* (Take-all fungus) and *Lomentospora prolificans*.

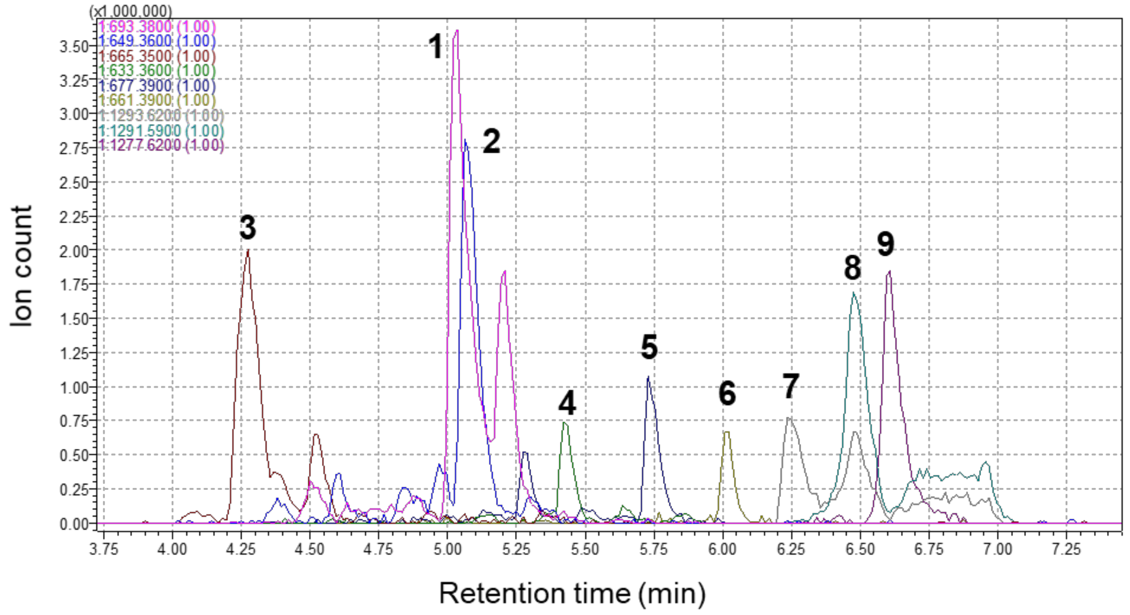

Peak number	Measured mass $[M+Na]^+$	Calculated mass $[M+Na]^+$	Δppm	Molecular Formula	Compound assignment
1	693.3842	670.3928	2.2	$C_{35}H_{58}O_{12}$	pentamycin
2	649.3585	626.3666	2.7	$C_{33}H_{54}O_{11}$	14-hydroxyisochainin
3	665.3491	642.3615	1.6	$C_{33}H_{54}O_{12}$	1´,14-dihydroxyisochainin
4	633.3624	610.3717	1.5	$C_{33}H_{54}O_{10}$	isochainin
5	677.3845	654.3979	2.6	$C_{35}H_{58}O_{11}$	filipin III
6	661.3959	638.4030	3.7	$C_{35}H_{58}O_{10}$	filipin II
7	1293.6184	1270.6234	5.8	$C_{62}H_{86}N_{12}O_{17}$	actinomycin $X_{0\beta}$
8	1291.5936	1268.6077	3.4	$C_{62}H_{84}N_{12}O_{17}$	actinomycin X_2
9	1277.6192	1254.6285	1.5	$C_{62}H_{86}N_{12}O_{16}$	actinomycin D

Figure S4. Ion chromatograms from UPLC-MS analysis of the crude ethyl acetate extract from *Streptomyces* strain N2 grown on SFM agar plates. Numbers above the peaks on the chromatogram represent the different compounds identified in the extract. The main compounds present are summarized in the table beneath the chromatogram.

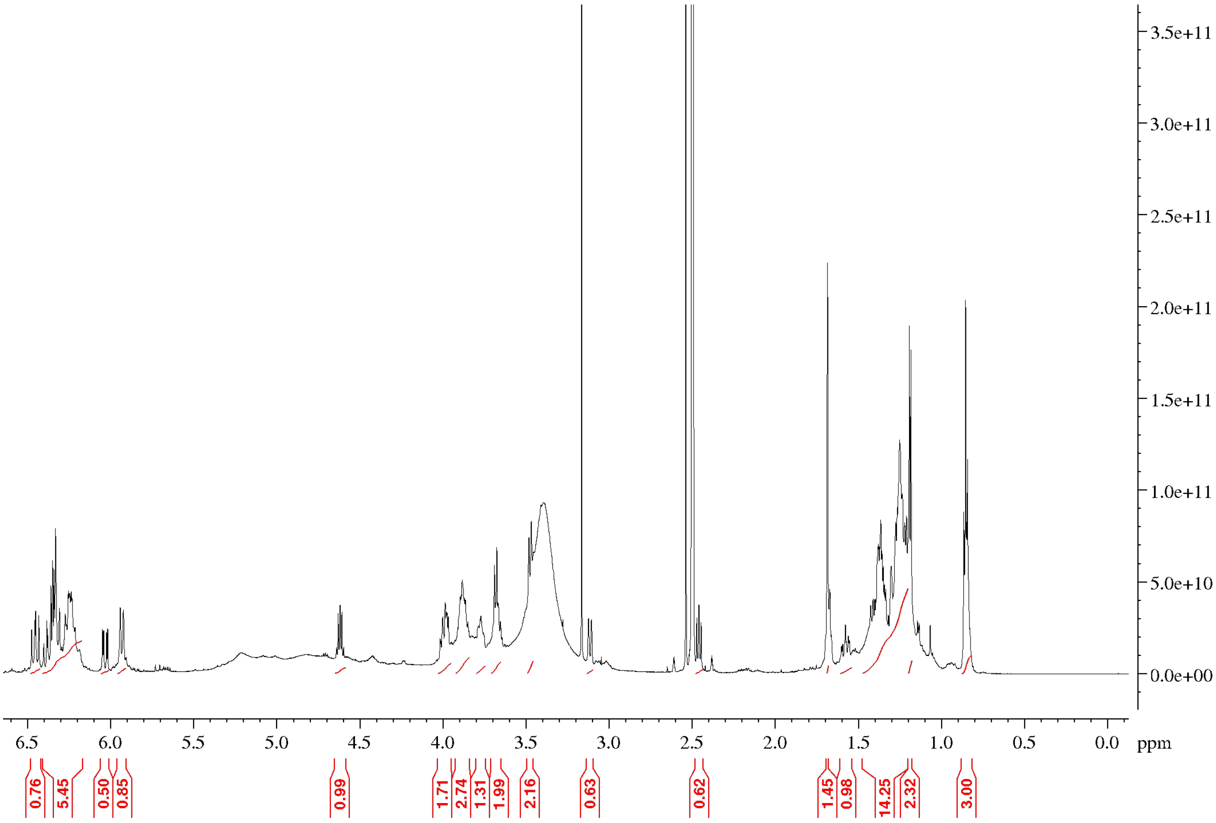

Figure S5. ^{1}H NMR spectrum of pentamycin at 600 MHz in DMSO-d_6.

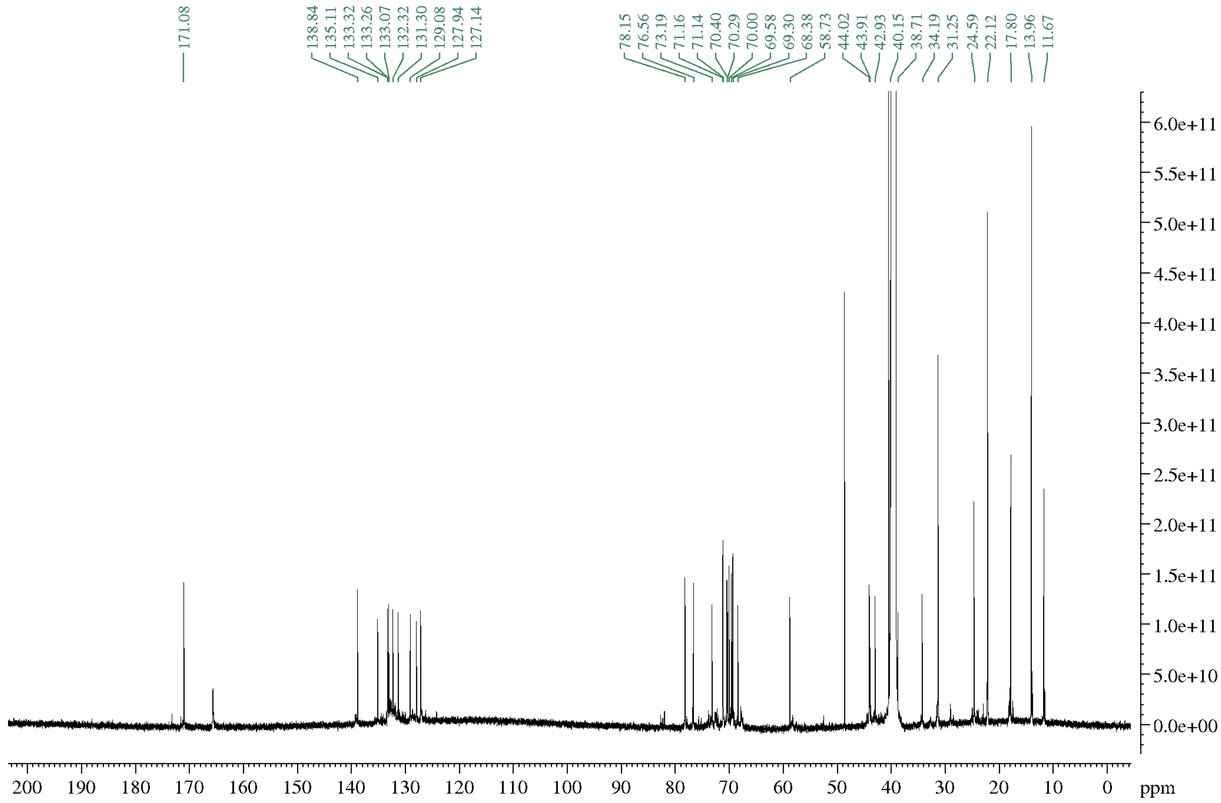

Figure S6. ^{13}C NMR spectrum of pentamycin at 150 MHz in DMSO-d_6.

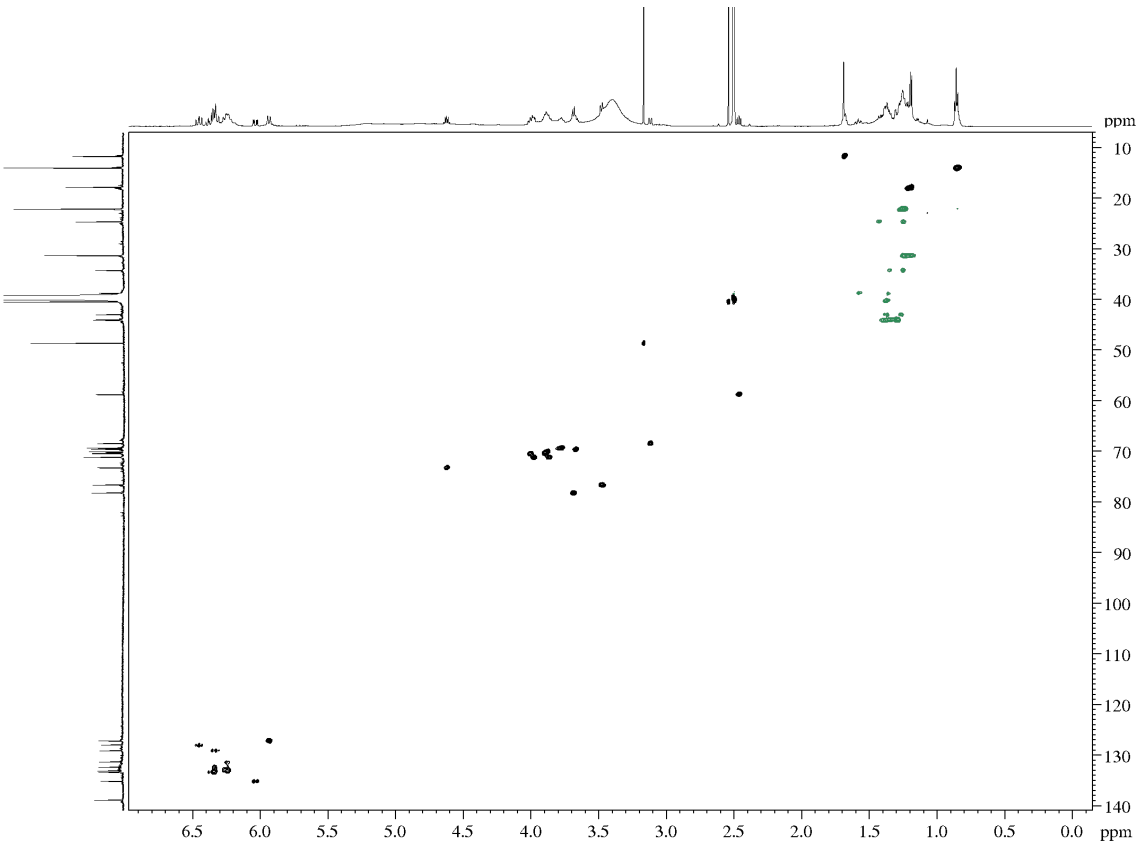

Figure S7. HSQCed spectrum of pentamycin in DMSO-d_6.

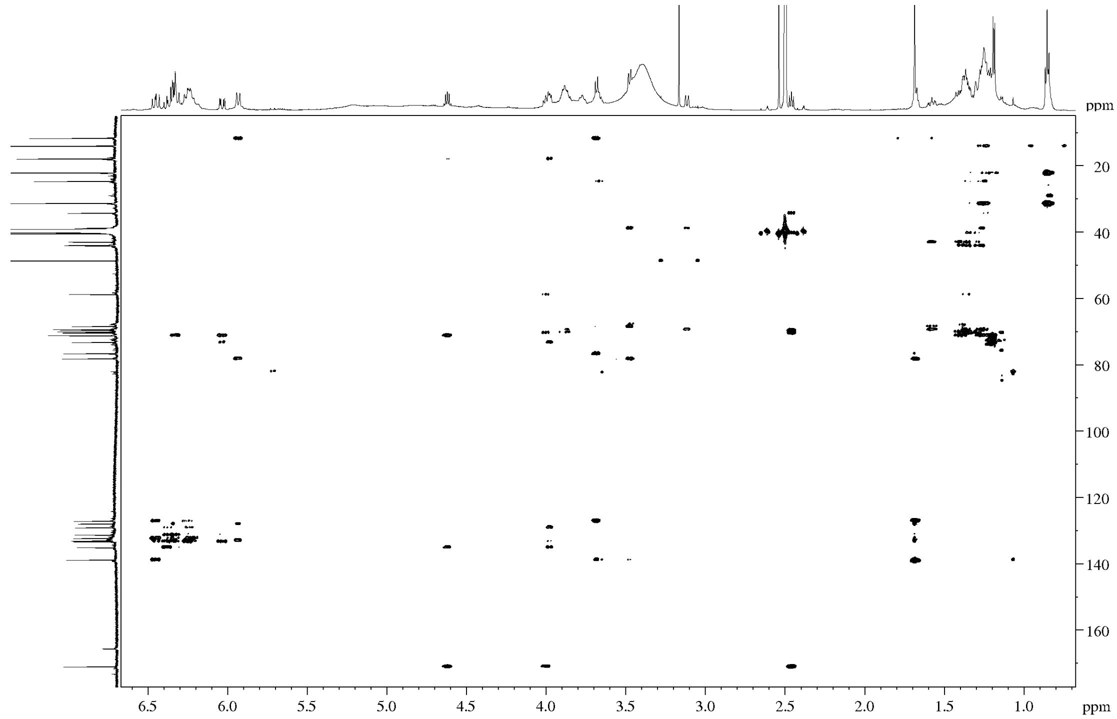

Figure S8. HMBC spectrum of pentamycin in DMSO-d_6

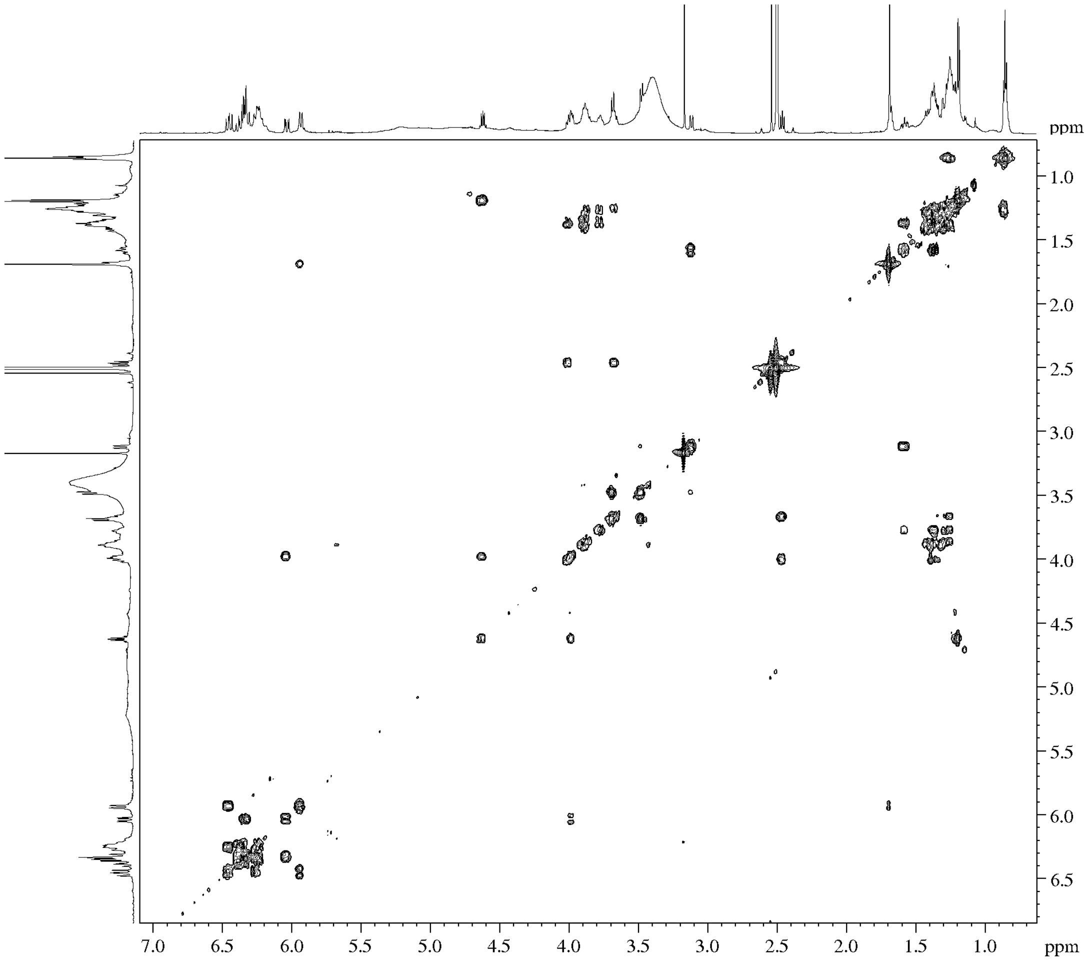

Figure S9. COSY spectrum of pentamycin in DMSO-d_6.

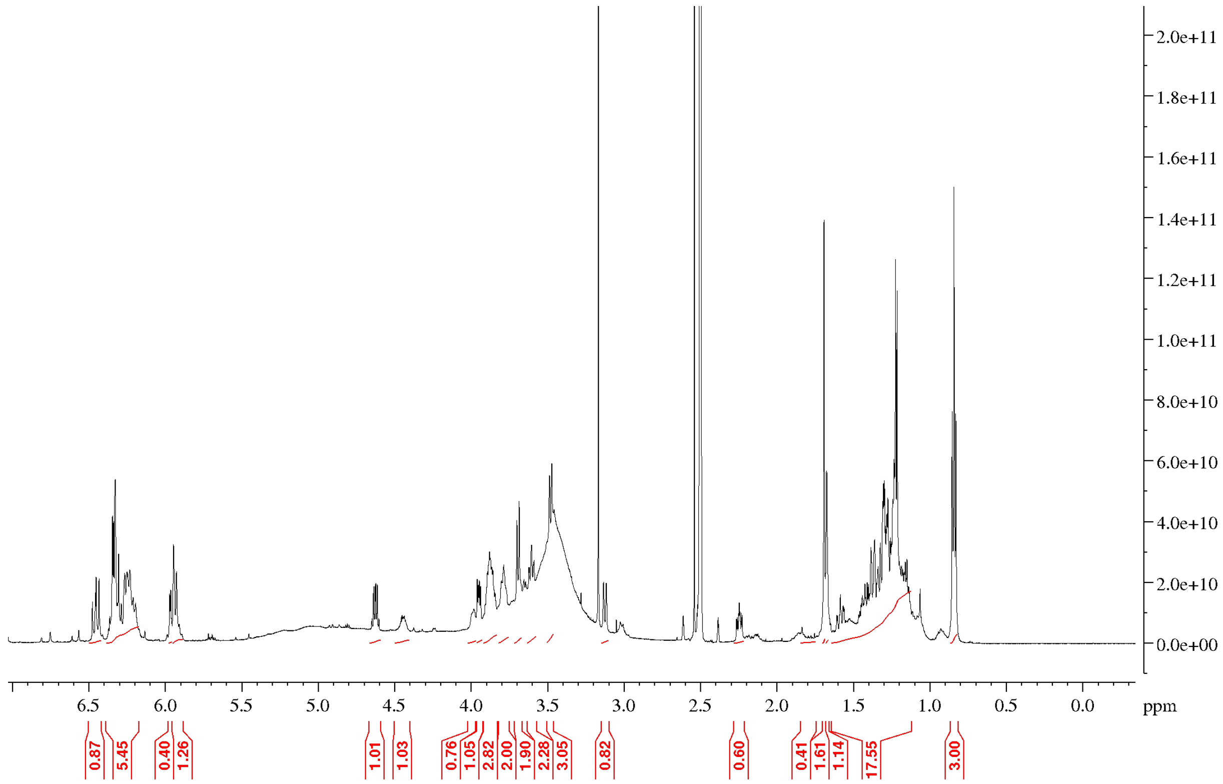

Figure S10. ^{1}H NMR spectrum of 14-hydroxyisochainin at 600 MHz in DMSO-d_6.

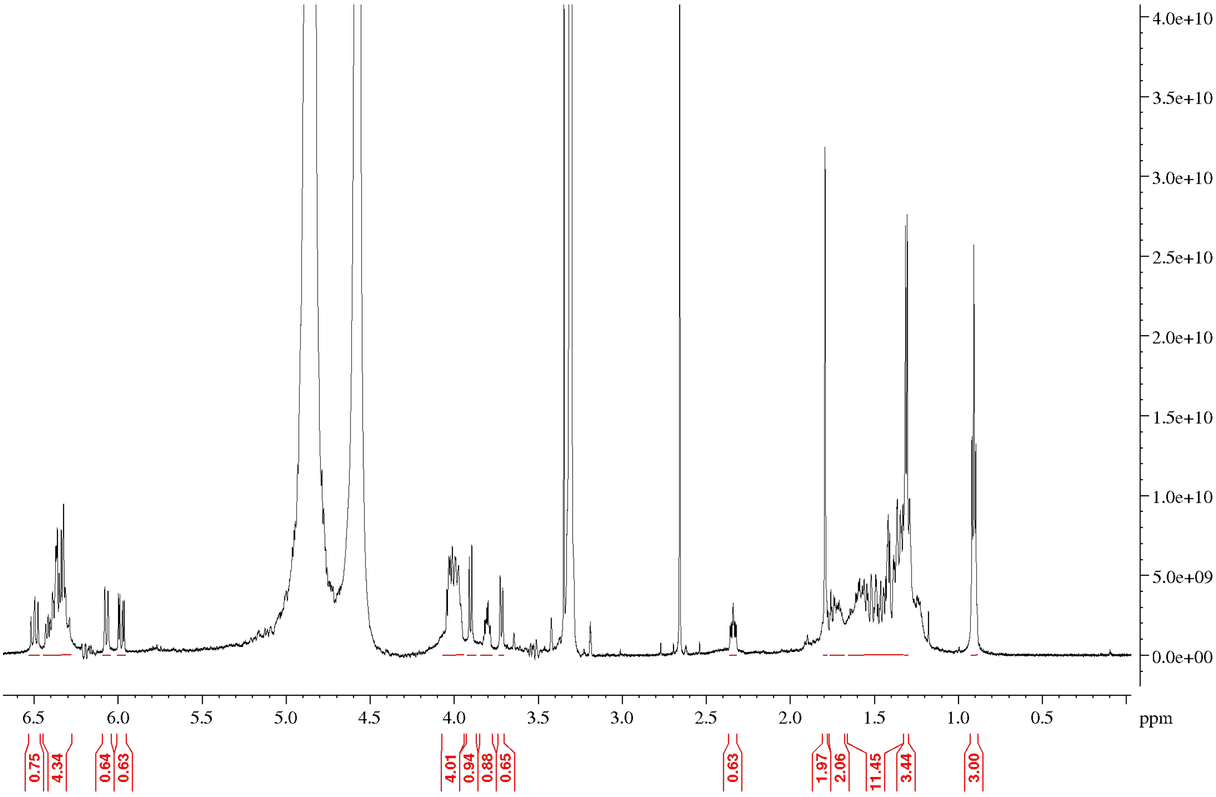

Figure S11. ^{1}H NMR spectrum of 14-hydroxyisochainin at 600 MHz in CD_3OD.

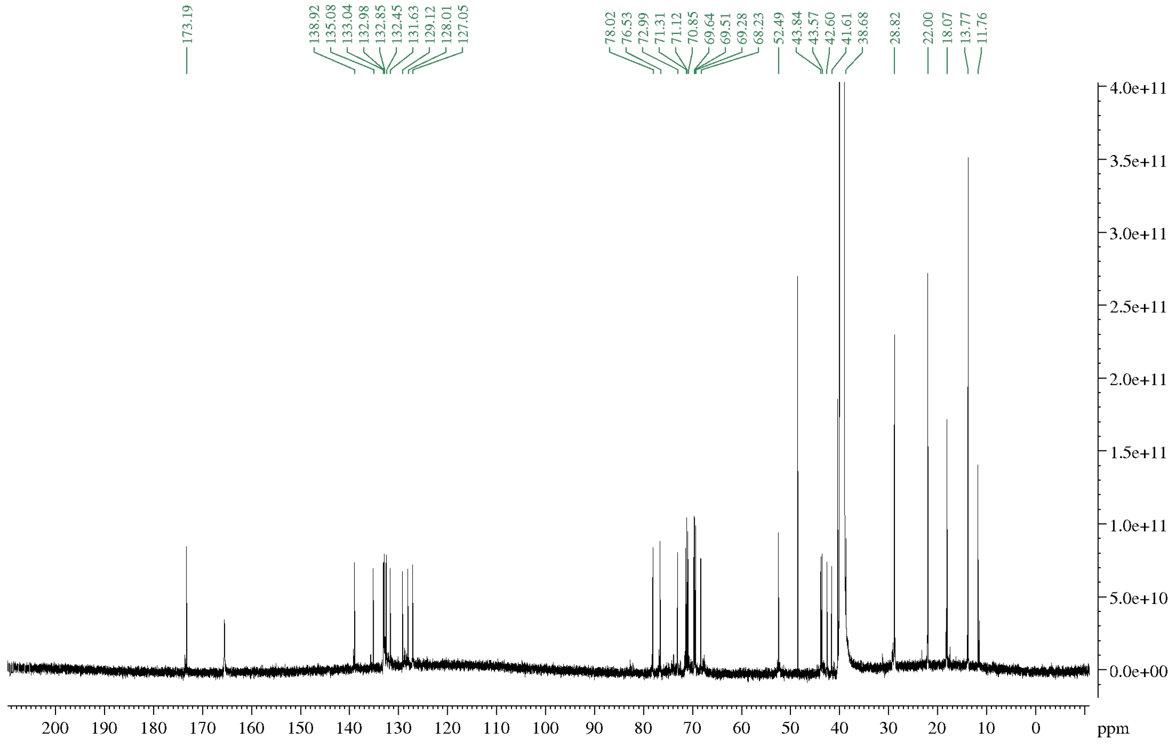

Figure S12. ^{13}C NMR spectrum of 14-hydroxyisochainin at 150 MHz in DMSO-d_6.

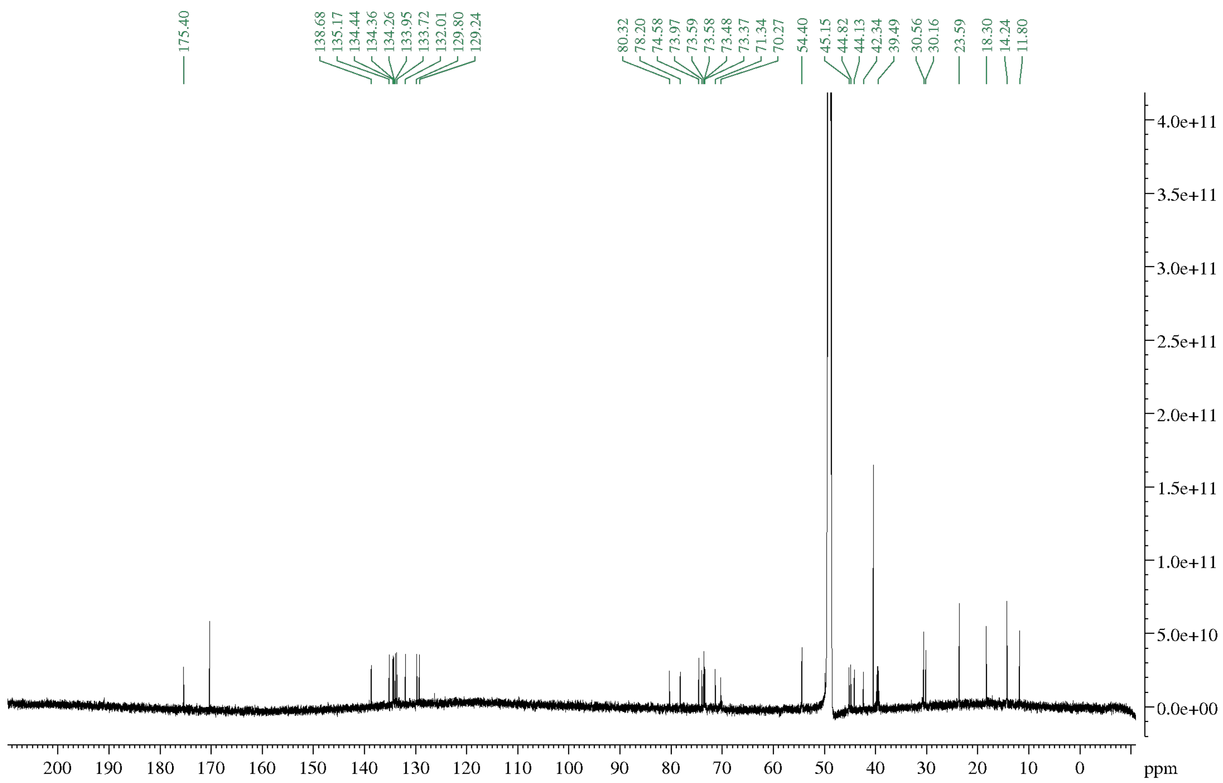

Figure S13. ^{13}C NMR spectrum of 14-hydroxyisochainin at 150 MHz in CD_3OD.

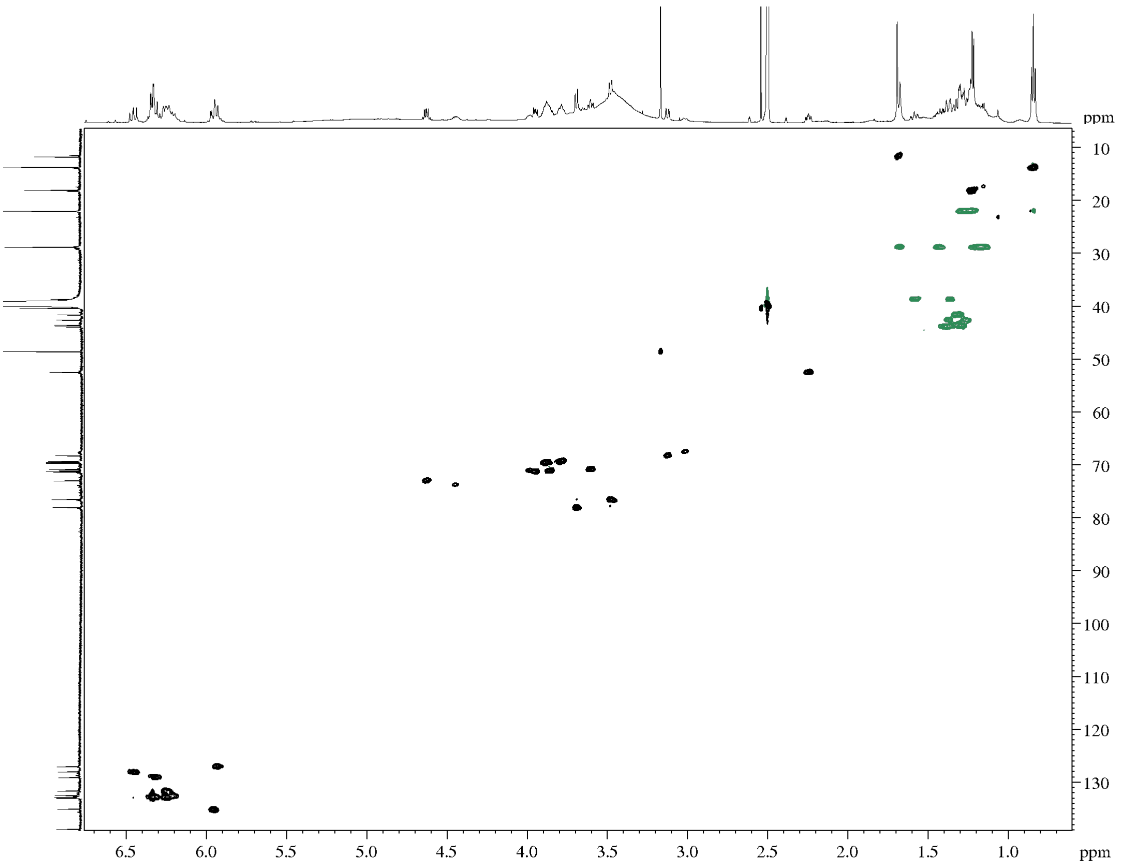

Figure S14. HSQCed spectrum of 14-hydroxyisochainin in DMSO-d_6.

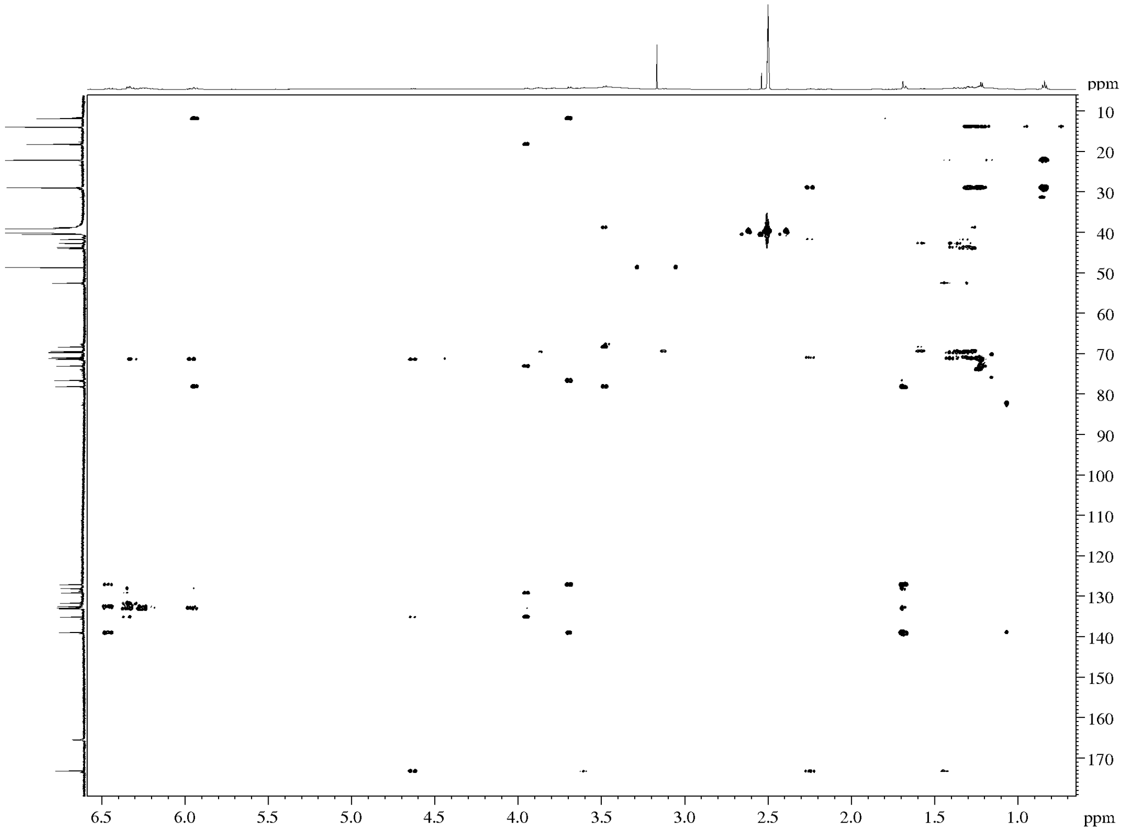

Figure S15. HMBC spectrum of 14-hydroxyisochainin in DMSO-d_6.

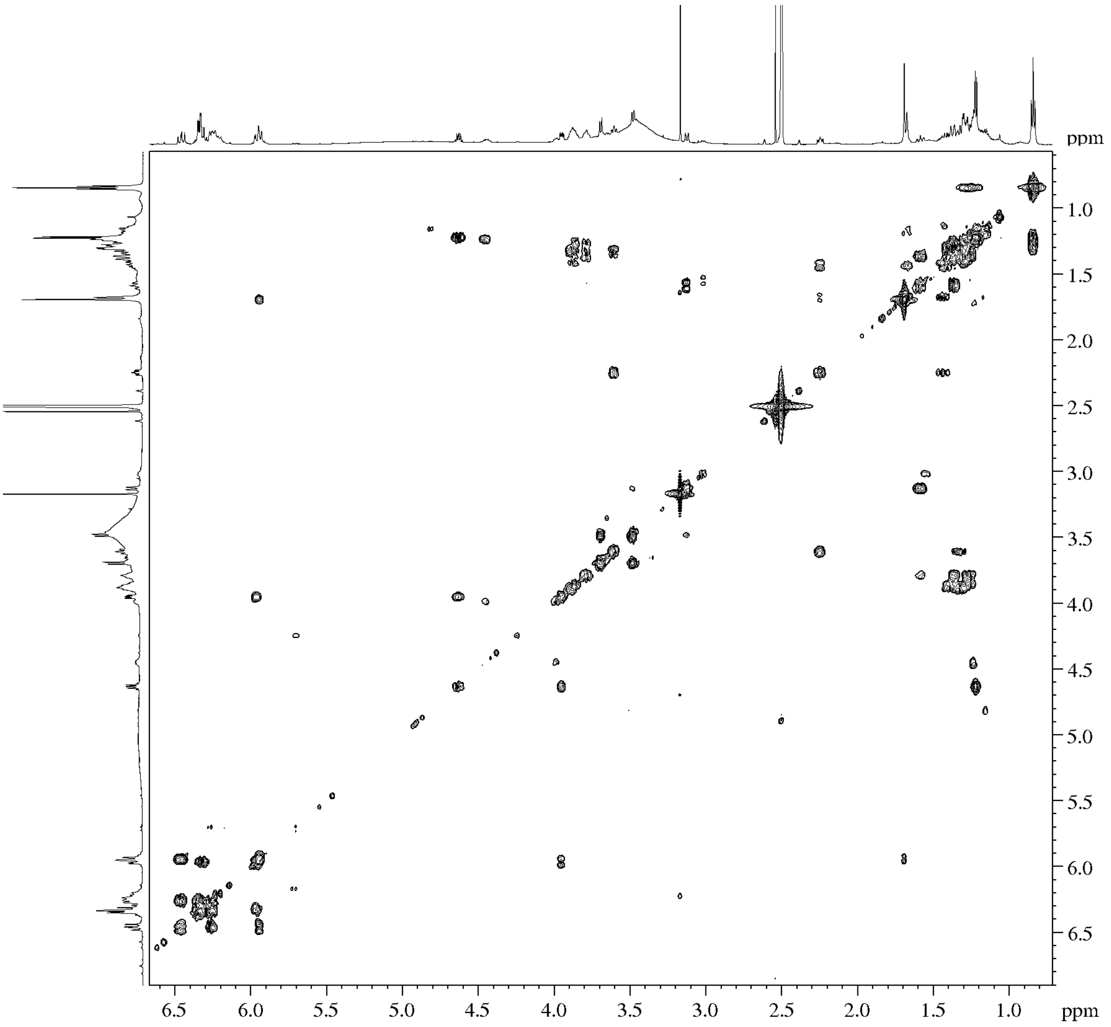

Figure S16. COSY spectrum of 14-hydroxyisochainin in DMSO-d_6.

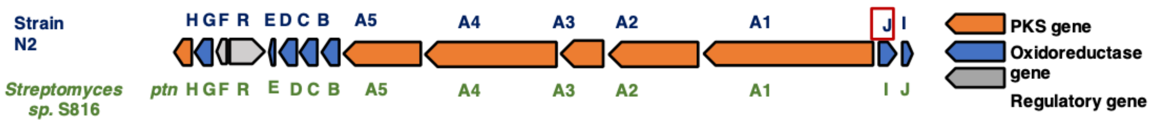

N2 Protein	*Streptomyces sp.* S816	Percentage identity (%)	Proposed function
I	ptnI	98	Ferredoxin for PtnJ
J	PtnJ	100	Cytochrome P450 hydroxylase
A1	ptnA1	94	PKS
A2	ptnA2	95	PKS
A3	ptnA3	95	PKS
A4	ptnA4	96	PKS
A5	ptnA5	96	PKS
B	ptnB	93	Putative dehydrogenase
C	ptnC	99	Cytochrome P450 hyrdoxylase
D	ptnD	98	Cytochrome P450 hyrdoxylase
E	ptnE	94	Ferrodoxin
R	ptnR	91	DnRI/RedD/AfsR-family transcriptional regulator
F	PtnF	95	LuxR family transcriptional regulator
G	PtnG	99	Putative oxidase
H	PtnH	97	Thioesterase

Figure S17. A comparison of the architecture of the biosynthetic gene cluster encoding filipin-like compounds in *Streptomyces* N2 and *Streptomyces* sp. S816 (1), respectively. Filipin biosynthesis is encoded by 13 genes (A1-H) and pentamycin is formed as a result of the hydroxylation of filipin III by an additional cytochrome P450 monooxygenase (J, outlined in red), the gene for which is located upstream of the main filipin cluster.

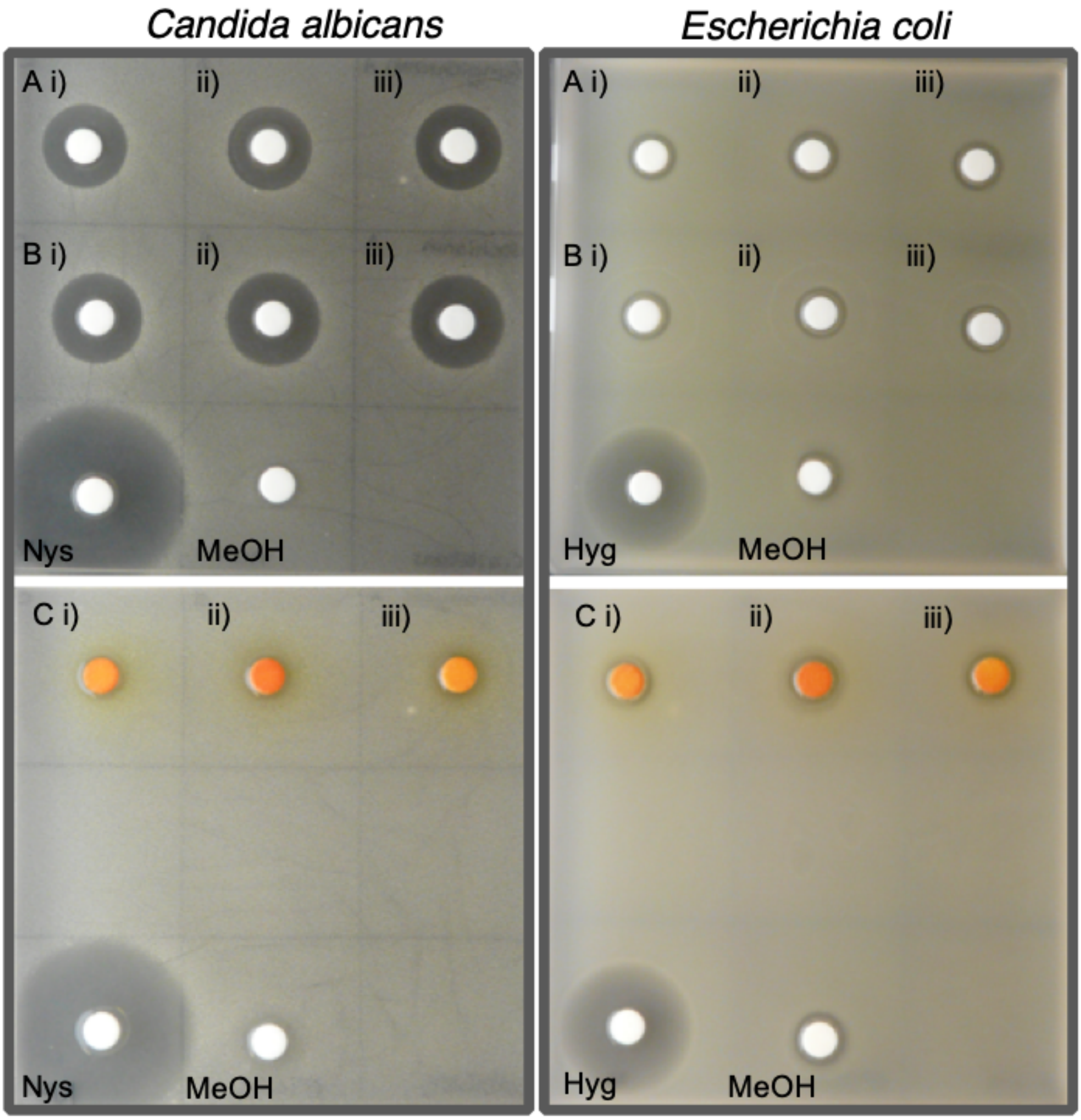

Figure S18. Disc-diffusion bioassays using compounds purified from the *Streptomyces* strain N2 against *Candida albicans* and *Escherichia coli*. Discs were soaked in purified extracts of either A) pentamycin, B) 14-hydroxyisochainin or C) actinomycin complex. N=3 (i-iii) technical replicates of each extract. Nystatin (Nys) or hygromycin (Hyg) were used as positive controls and methanol (MeOH) was used as a negative control.

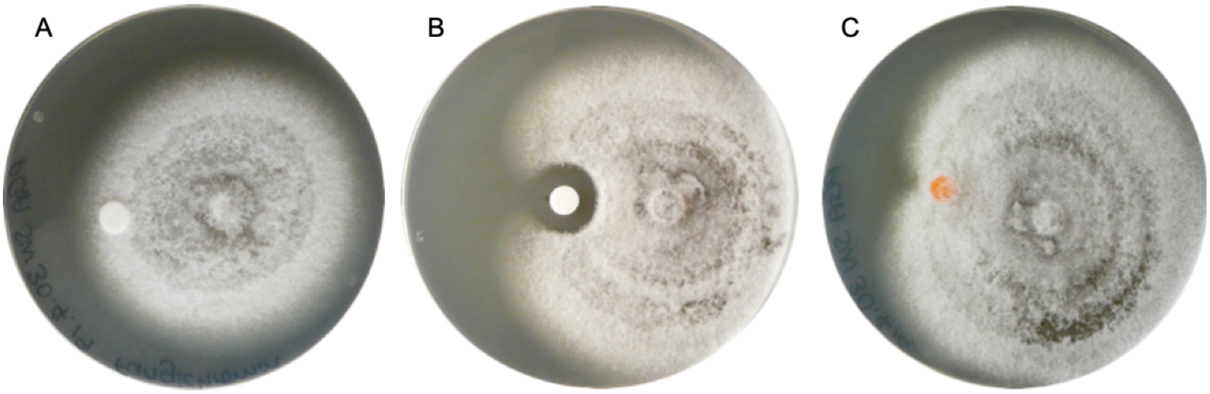

Figure S19. Disc-diffusion bioassays using compounds purified from *Streptomyces* strain N2 against *G. graminis* var. *tritici* (Take-all fungus). Discs were soaked in purified extracts of either: A) pentamycin: B) 14-hydroxyisochainin; or C) actinomycin complex. Antifungal activity is indicated by a zone of clearing around the disc.

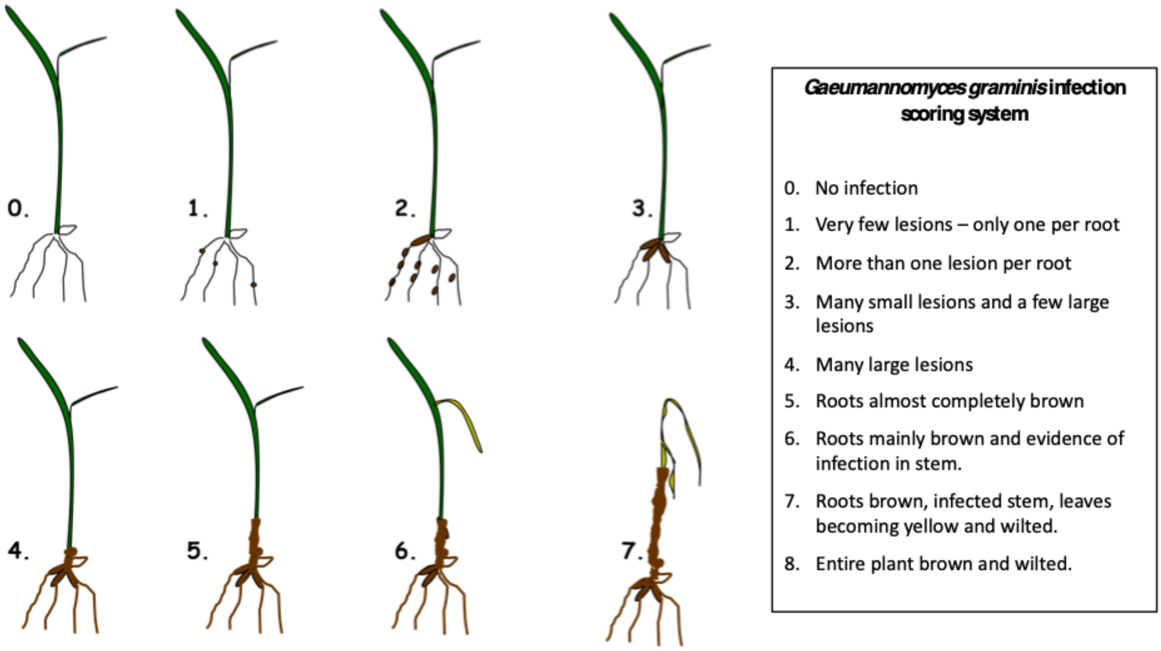

Figure S20. Infection scoring system for the vermiculite-based Take-all infection assay of *Triticum aestivum.*

Supplementary tables

Table S1. The presence and absence of genes encoding enzymes involved in plant growth promotion pathways in sequenced *Streptomyces* isolates. Green indicates the presence of the enzyme in a given isolate, red indicates the absence of the enzyme.

**Pathway**	**Enzyme**	**KO number**	**Kegg Reaction**	***Streptomyces* strain**								
				**MG**	**M2**	**M3**	**N1**	**N2**	**L2**	***S. lydicus* 25470**	***S. lydicus* 31975**	**Actinovate**
IAA biosynthesis: IpyA pathway	Tryptophan aminotransferase	K14265	R00684									
	Indole-3-pyruvate decarboxylase	K04103	R01974									
	Acetaldehyde oxidase/ dehydrogenase	K11817/ K00128	R02681/ R02678									
IAA biosynthesis: IAM pathway	Tryptophan 2-monooxygenase (IaaM)	K00466	R00679									
	IAM hydrolase (IaaH)	K01426/ K21801	R03096									
IAA biosynthesis: IAN pathway	Nitrilase	K01501	R03093									
	Tryptophan decarboxylase	K01593	R00685									

IAA biosynthesis: TAM pathway	(Mono or di) Amine oxidase	K00274/ K11182	R02173									
	Acetaldehyde oxidase/ dehydrogenase	K11817/ K00128	R02681/ R02678									
Regulation of plant ethylene production	1-aminocyclopropane-1-carboxylic acid (ACC) deaminase	K01505	R00997									

Table S3. Bioactivity screens of plant-associated *Streptomyces* species (purple columns, top) against a range of different indicator species (blue column, down). Screens were conducted on a range of different media (see Table S5 for recipes). Green squares indicate inhibition of the indicator strain by the given streptomycete, red indicates no inhibition.

**Pathogen strain**	**Media**	**Streptomycete strain**							
		**N1**	**N2**	**M3**	**M2**	**L2**	***S. lydicus* 25470**	***S. lydicus* 31975**	**Actinovate**
Bacillus subtilis	SFM								
	Minimal salts								
	ISP2								
	Oatmeal								
	MYM								
Methicillin-resistant Staphylococcus aureus	SFM								
	Minimal salts								
	ISP2								
	Oatmeal								
	MYM								
Escherichia coli	SFM								
	minimal salts								
	ISP2								
	Oatmeal								
	MYM								
Pseudomonas syringae DC3000	SFM								
	Minimal salts								
	ISP2								
	Oatmeal								
	MYM								
	SFM								

Candida albicans	Minimal salts								
	ISP2								
	PGA								
	Oatmeal								
	MYM								
Gaeumannomyces graminis	PGA								
Lomentospora prolificans	PGA								

Table S4. NMR shift data for pentamycin; 600/150 MHz in DMSO-d_6. *Signals may interchange due to resonance.

	^{13}C	^{1}H (HSQC)	HMBC	COSY
1	171.08		2;3;27	
16	138.84		15;8;29	
25	135.11	6.04 (dd, J=14.6 Hz; 4.5 Hz)	23;24;26; 27	24;26
19-23*	133.32	6.40 (m)		
19-23*	133.26	6.34 (m)		
19-23*	133.07	6.25 (m)		
19-23*	132.32	6.26 (m)		
19-23*	131.30	6.25 (m)		
24	129.08	6.34 (m)	26	25
18	127.94	6.46 (dd, J=14.5 Hz, 11.4 Hz)	17;29	17
17	127.14	5.94 (d, J=11.2 Hz)	15;18;29	18
15	78.15	3.69 (d, J=8.9 Hz)	14;17;29	14
14	76.56	3.48 (dd, J=8.9 Hz, 1.3 Hz)	15	13;15
27	73.19	4.62 aqd (J=7.3 Hz, 6.4 Hz)	25;26;28	26;28
26	71.16	3.98 (m)	24;25;27; 28	25;27
9	71.14	3.86 (m)	8; 10	8;10
3	70.40	4.00 (m)	2;4	2;4
7	70.29	3.89 (m)	6; 8	6;8
5	70.00	3.89 (m)	4	4;6
1´	69.58	3.67 (m)	2	2;2´
11	69.30	3.77 (m)	9;10;12;1 3	10;12
13	68.38	3.12 (d, J=10.7 Hz)	14	12;14
2	58.73	2.46 (dd, J=8.5 Hz, 7.4 Hz)	1´;2´;3;4	1´;3
8	44.02 (CH_2)	1.39 (m), 1.28 (m)	6;10	7;9
6	43.91 (CH_2)	1.34 (m), 1.30 (m)	4;8	5;7

10	42.93 (CH_2)	1.36 (m), 1.26 (m)	8;12	9;11
4	40.15 (CH_2)	1.38 (m)	2;6	3;5
12	38.71 (CH_2)	1.58 (m), 1.36 (m)	10;11;13; 14	11;13
2´	34.19 (CH_2)	1.35 (m), 1.25 (m)	1´;2;3´;4´	1´
4´	31.25 (CH_2)	1.25 (m), 1.19 (m)	2´;3´;5´;6´	
3´	24.59 (CH_2)	1.43 (m), 1.25 (m)	1´;2´;4´	
5´	22.12 (CH_2)	1.26 (m)	4´;6´	6´
28	17.80	1.19 (d, J=6.3 Hz)	26;27	27
6´	13.96	0.85 (t, J=7.0 Hz)	5´	5´
29	11.67	1.69 (s)	15;17	17

Table S5. NMR data for 14-hydroxyisochainin; 600/150 MHz in DMSO-d_6. *Signals can interchange due to resonance; **tentative.

Position	^{13}C	^1H (HSQC)	HMBC	COSY
**1**	173.19		1´;2;3;27	
**16**	138.92		15;18;29	
**25**	135.08	5.96 (m)	24;26;27	24;26
**19-23***	133.04	6.32 (m)		
**19-23***	132.98	6.32 (m)		
**19-23***	132.85	6.32 (m)		
**19-23***	132.45	6.26 (m)		
**19-23***	131.63	6.25 (m)		
**24**	129.12	6.33 (m)	26	23;25
**18**	128.01	6.46 (dd, J=11.7, 11.1 Hz);	17;29	17
**17**	127.05	5.94 (m)	15;18;29	18;29
**15**	78.02	3.69 (d, J=8.8 Hz)	14;17;29	14
**14**	76.53	3.48 (dd, J=8.9; 1.2 Hz)	15	13;15
**27**	72.99	4.63 (m)	26;28	26;28
**26**	71.31	3.95 (dd, J= 8.4 Hz; 4.7 Hz)	24;25;27;28	25;27
**9**	71.12	3.86 (m)	8;10	
**3**	70.85	3.61 (m)	2;4	2;4
**7****	69.64	3.88 (m)	6;8	6
**5****	69.51	3.88 (m)	4;6	6
**11**	69.28	3.79 (m)	10;12;13	10;12
**13**	68.23	3.13 (d, J=10.8 Hz)	12;14	12;14
**2**	52.49	2.25 (m)	1´;2´;3;4	1´;3
**8**	43.84 (CH_2)	1.39 (m)	6;10	7
**6**	43.56 (CH_2)	1.31 (m)	4;8	5;7
**10**	42.60 (CH_2)	1.37 (m), 1.27 (m)	8;12	11
**4**	41.61 (CH_2)	1.31 (m)	2;6	3
**12**	38.68 (CH_2)	1.58 (m), 1.36 (m)	10;14	11;13
**1´**	28.82 (CH_2)	1.67 (m), 1.43 (m)	2;2´;3;3´;	2;2´

**2´**	28.82 (CH_2)	1.17 (m)	1´;2;3´;4´	1´
**3´**	22.00 (CH_2)	1.26 (m)	2´;4´	4´
**28**	18.07	1.22 (d, J=6.4 Hz)	26	27
**4´**	13.77	0.84 (t, J=7.2 Hz)	2´; 3´	3´
**29**	11.76	1.69 (s)	17	

Table S6. Comparison of ^{13}C chemical shifts measured at 600 MHz in CD_3OD for 14-hydroxyisochainin isolated in this study and reported in Li *et al* (2).

^{13}C shifts this study	Position	^{13}C shifts Li *et al*
175.40	1	175.37
138.68	16	138.71
135.17	19	135.18
134.44	21	134.45
134.36	25	134.37
134.26	23	134.32
133.95	20	133.96
133.72	22	133.74
132.01	24	131.99
129.80	17	129.79
129.24	18	129.25
80.32	15	80.32
78.20	14	78.20
74.58	27	74.58
73.97	9	74.02
73.59	5	73.64
73.58	7	73.56
73.48	3	73.55
73.37	26	73.44
71.34	11	71.35
70.27	13	70.26
54.40	2	54.40
45.15	8	45.16
44.82	6	44.83
44.13	10	44.15
42.34	4	42.33
39.49	12	39.50
30.56	1´	30.57

30.16	2´	30.18
23.59	3´	23.61
18.30	28	18.30
14.24	4´	14.25
11.80	29	11.80

Table S7. Media recipes used in experiments

Media	Component	g L^{-1} dH_2O
Soya Flour Mannitol (SFM) agar (3)	Soy flour	20
	Mannitol	20
	Agar	20
Minimal Salts medium (4)	NH_4SO_4	2
	K_2HPO_4	14
	KH_2PO_4	6
	Sodium citrate	1
	$MgSO_4$	0.2
	Agar	15
Maltose-Yeast extract-Malt extract (MYM) agar	Maltose	4
	Yeast Extract	4
	Malt Extract	10
	Agar	18
ISP2 Agar	Yeast Extract	4
	Maltose	10
	D-glucose	4
	Agar	20
Oatmeal Agar	Ground oats	20
	Agar	20
Potato Glucose Agar (PGA)	PGA (Sigma Aldrich)	39
Lysogeny Broth (LB)	Tryptone	10
	NaCl	10
	Yeast extract	5
	Glucose (only for growth of *P. syringae*)	1
	NH_4SO_4	1
	KH_2PO_4	0.5
	$MgSO_4.7H_2O$	0.2
	$FeSO_4.H_2O$	0.01

Minimal medium ± IAA (3)	Agar	15
	Trace elements (added after autoclaving)	2 ml
	± Indole Acetic Acid (IAA, Sigma Aldrich)	0.1
Dworkins and Foster medium (5)	$(NH_4)_2SO_4$	2
	KH_2PO_4	4
	Na_2HPO_4	6
	$MgSO_4.7H_2O$	0.2
	$FeSO_4.H_2O$	0.001
	H_3BO_4	0.0001
	$MnSO_4$	0.0001
	$ZnSO_4$	0.0007
	$CuSO_4$	0.0005
	MoO_3	10
	Agar	20
2xYT	Bacto-tryptone	16
	Yeast extract	10
	NaCl	5
Murashige and Skoog (MSk) Agar	Murashige and Skoog salts (Duchefa Biochemie, Harlem, Netherlands)	4.43
	Sucrose	10 or 0
	Agar	8 or 15
Yeast Peptone Dextrose (YPD) Agar	Yeast Extract	10
	Bactopeptone	40
	Glucose	15
	Agar	15
Silwett L-77 amended Phosphate Buffered Saline (PBS-S)	$NaH_2PO_4.H_2O$	6.33 g
	$Na_2HPO_4.H_2O$	16.5 g
	200 µl Silwet L-77 added after autoclaving	

References

1) Frey Tirri B, Bitzer J, Geudelin B, Drewe J. 2010. Safety, tolerability and pharmacokinetics of intravaginal pentamycin. Chemotherapy 56:190-6.
2) Li Z, Rawlings BJ, Harrison PH, Vederas JC. 1989. Production of new polyene antibiotics by *Streptomyces cellulosae* after addition of ethyl (Z)-16-phenylhexadec-9-enoate. J Antibiot (Tokyo) 42:577-84.
3) Kieser T, Bibb MJ, Buttner MJ, Chater KF, Hopwood DA. 2000. Practical *Streptomyces* Genetics. John Innes Foundation, Norwich.
4) Lebeis SL, Paredes SH, Lundberg DS, Breakfield N, Gehring J, McDonald M, Malfatti S, Del Rio TG, Jones CD, Tringe SG. 2015. Salicylic acid modulates colonization of the root microbiome by specific bacterial taxa. Science 349:860-864.
5) Dworkin M, Foster JW. 1958. Experiments with some microorganisms which utilize ethane and hydrogen. J Bacteriol 75:592-603.
